# Multiparameter Flow Cytometry Analysis of the Human Spleen Applied to Studies of Plasma-Derived EVs From *Plasmodium vivax* Patients

**DOI:** 10.3389/fcimb.2021.596104

**Published:** 2021-03-01

**Authors:** Melisa Gualdrón-López, Míriam Díaz-Varela, Haruka Toda, Iris Aparici-Herraiz, Laura Pedró-Cos, Ricardo Lauzurica, Marcus V. G. Lacerda, Marco Antonio Fernández-Sanmartín, Carmen Fernandez-Becerra, Hernando A. del Portillo

**Affiliations:** ^1^ISGlobal, Hospital Clinic - Universitat de Barcelona, Barcelona, Spain; ^2^IGTP: Germans Trias i Pujol Research Institute, Barcelona, Spain; ^3^Nephrology Service, Germans Trias i Pujol University Hospital, Badalona, Spain; ^4^Fundaçao de Medicina Tropical Dr. Heitor Vieira Dourado, Manaus, Brazil; ^5^Instituto Leônidas & Maria Deane (ILMD), Fiocruz, Manaus, Brazil; ^6^ICREA: Catalan Institution for Research and Advanced Studies, Barcelona, Spain

**Keywords:** *Plasmodium vivax*, human spleen, extracellular vesicles, multiparameter flow cytometry, interaction

## Abstract

The spleen is a secondary lymphoid organ with multiple functions including the removal of senescent red blood cells and the coordination of immune responses against blood-borne pathogens, such as malaria parasites. Despite the major role of the spleen, the study of its function in humans is limited by ethical implications to access human tissues. Here, we employed multiparameter flow cytometry combined with cell purification techniques to determine human spleen cell populations from transplantation donors. Spleen immuno-phenotyping showed that CD45^+^ cells included B (30%), CD4^+^ T (16%), CD8^+^ T (10%), NK (6%) and NKT (2%) lymphocytes. Myeloid cells comprised neutrophils (16%), monocytes (2%) and DCs (0.3%). Erythrocytes represented 70%, reticulocytes 0.7% and hematopoietic stem cells 0.02%. Extracellular vesicles (EVs) are membrane-bound nanoparticles involved in intercellular communication and secreted by almost all cell types. EVs play several roles in malaria that range from modulation of immune responses to vascular alterations. To investigate interactions of plasma-derived EVs from Plasmodium vivax infected patients (PvEVs) with human spleen cells, we used size-exclusion chromatography (SEC) to separate EVs from the bulk of soluble plasma proteins and stained isolated EVs with fluorescent lipophilic dyes. The integrated cellular analysis of the human spleen and the methodology employed here allowed in vitro interaction studies of human spleen cells and EVs that showed an increased proportion of T cells (CD4+ 3 fold and CD8+ 4 fold), monocytes (1.51 fold), B cells (2.3 fold) and erythrocytes (3 fold) interacting with PvEVs as compared to plasma-derived EVs from healthy volunteers (hEVs). Future functional studies of these interactions can contribute to unveil pathophysiological processes involving the spleen in vivax malaria.

## Introduction

The spleen is a secondary lymphoid organ with multiple functions in physiology and immunity, including the removal of senescent red blood cells (RBCs) from circulation, the recycling of iron and the coordination of innate and adaptive immune responses against blood-borne pathogens (Bowdler, 2002; Mebius and Kraal, 2005). Functionally, the spleen is organized in two distinct compartments: i) the white pulp, which is responsible for initiation of adaptive immune responses against blood pathogens and is composed by B cell and T cell zones; ii) the red pulp, which contains neutrophils, monocytes, dendritic cells (DCs), γδ T cells and macrophages, and its functions include the monitoring of aged, dead or opsonized RBCs as well as pathogen surveillance (Lewis et al., 2019). The spleen is involved in the control of bacterial, viral, fungal and parasitic infections, including malaria (Bronte and Pittet, 2013), a world threatening infectious disease caused by several species of the genus *Plasmodium* spp. In 2018, this disease registered 228 million cases and 405,000 deaths globally (World Health Organization, 2019).

Despite the fundamental role of the spleen, most of our current understanding of its structure and function in humans comes from extrapolations from rodent species. Animal models enable more convenient access to tissue samples and have allowed to investigate the spleen using techniques such as multiparameter flow cytometry in a great variety of physiological and pathological conditions (Steiniger, 2015). Comparisons between mice and human spleen architecture have shown similarities between species but also have revealed important structural differences, suggesting functional divergence requiring further investigation in the human spleen (Steiniger, 2015). The technical and ethical implications to perform such studies using human tissue have hampered our advance in this regard. Human spleen studies have been mostly performed in postmortem samples or biopsies of particular tissue sites using non-specific cellular architecture staining methods and single antigen characterization by immunohistochemistry (Lewis et al., 2019). Several studies of the immunological function of particular splenic cell types in humans have been reported (Buffet et al., 2006; Langeveld et al., 2006; Velásquez-Lopera et al., 2008; Petvises et al., 2016; Meinderts et al., 2017; Nagelkerke et al., 2018); however, to the best of our knowledge, no approaches have addressed the description of the whole spectrum of cells in the human spleen. A recent study using a large cohort of organ transplantation donors as source of lymphoid organs, including the spleen, has shown the most complete characterization of human spleen immune cells including myeloid populations as monocytes and neutrophils as well as lymphoid cells at different activation states (Carpenter et al., 2018a). These studies highlighted the relevance of organ transplantation donors as a source of physiological human tissue to conduct immunological studies.

Extracellular vesicles (EVs) are heterogeneous double membrane particles that have emerged as relevant mediators of intercellular communication and can be secreted by virtually every cell type (Théry et al., 2002b; Gho and Lee, 2017). EVs can be classified into two main categories, exosomes and microvesicles (MVs), based on their size, biogenesis and composition. Exosomes are 30–100 nm vesicles of endocytic origin that are released after the fusion of multivesicular bodies (MVBs) with the plasma membrane. MVs, also sometimes referred to as microparticles (MPs), have a more heterogeneous shape, can be bigger in diametrical size (1 µm) and are shed directly from the plasma membrane. Notably, exosomes and microvesicles have been reported to play several roles during malaria infections, including modulation of immune responses, promotion of development of sexual stages responsible for transmission, and alteration of vascular endothelium, among others (Regev-Rudzki et al., 2013; Marcilla et al., 2014; Mantel et al., 2016; Sampaio et al., 2017; Sisquella et al., 2017). Indirect associations of EV release with malaria pathology were originally observed in infections caused by *P. falciparum* and *P. vivax*, the two malaria species responsible for most of the burden, morbidity and mortality associated to human malaria (Campos et al., 2010; Nantakomol et al., 2011). Most studies of EVs in malaria, however, have been performed using EVs isolated from *in vitro* culture systems or experimental rodent infections. Thus, the physiological role of EVs in human malaria infections remains to be determined. Remarkably, using EVs obtained from circulating blood of *P. vivax* patients, we recently showed that they contain parasite proteins and are taken up by human spleen fibroblasts inducing expression of ICAM-1 *via* NF-kB and facilitating cytoadherence of *P. vivax*-infected reticulocytes obtained from patients (Toda et al., 2020).

Here, we report the first integrated characterization of human spleen cells using multiparameter flow cytometry describing subpopulations of splenic leukocytes and RBCs. We employed this methodology combined with cell purification techniques to address the interaction of plasma-derived EVs from *P. vivax* patients as opposed to healthy human volunteers with different spleen cell subpopulations.

## Methods

### Malaria Patients and Healthy Donors

Plasma samples from *P. vivax*-infected patients (PV) were collected at the Hospital of the Fundação de Medicina Tropical Doutor Heitor Vieira Dourado (Manaus, Amazonas, Brazil). The local ethical committee of FMT-HVD approved these studies. Clinical data of patients participating of this study has been recently published (Toda et al., 2020). Plasma from healthy donors (HD) was obtained at the Hospital Germans Trias i Pujol (Badalona, Barcelona, Spain) after expressed consent from the donors.

### Human Spleen Donors

Human spleens used in this study were retrieved from deceased transplantation donors at the Hospital Germans Trias i Pujol (Badalona, Barcelona, Spain). Donors comprised 57% of men and 43% women aged 25–66 years old. Cause of death of transplantation donors is described in **Supplementary Data Sheet 1**. Donation of these organs for biomedical research received written consent from family members and was in accordance with the protocol approved by the Ethics Committee for Clinical Research of the Hospital Germans Trias i Pujol.

### Blood Collection and Plasma Processing for Extracellular Vesicles Isolation

Three mL of peripheral blood were collected by venipuncture in citrate pre-treated tubes. Samples were centrifuged at 400 xg for 10 min at RT. Plasma was collected and centrifuged at 2,000 xg for 10 min at 4°C. Supernatant was recovered, aliquoted and frozen at -80°C. Frozen plasmas were shipped from malaria endemic regions to IGTP (Badalona, Spain). Plasma collected from HD was similarly processed.

### Extracellular Vesicles Purification by Size-Exclusion Chromatography

EVs were isolated from plasma samples of either *P. vivax*-infected patients or healthy donors by size-exclusion chromatography as previously described (de Menezes-Neto et al., 2015). Briefly, plasma was defrosted on ice, centrifuged twice at 2,000 xg for 10 min at 4°C to pellet debris. 1 mL of supernatant was loaded on the top of 10 mL-sepharose CL-2B (Sigma) that had been packed in a syringe and pre-equilibrated with 1X PBS. 15 fractions of 500 µL were collected immediately after loading of plasma, aliquoted and frozen at —80°C until use. Protein concentration of chromatographic fractions was measured by BCA assay (Thermo Scientific). The whole purification procedure was performed in sterile conditions.

### Bead-Based Flow Cytometry Analysis of Extracellular Vesicles

EV-enriched SEC fractions were identified and molecularly characterized by bead-based flow cytometry (Théry et al., 2006). 45 µL of SEC fractions were coupled to 5 µL of 1:10 pre-diluted solution of 4 µm-aldehyde/sulfate-latex beads (Invitrogen). PBS was added to the negative-control tubes. Coupling incubation was performed for 15min at RT. Beads were then resuspended in 1 mL of bead-coupling buffer (BCB: PBS with 0.1% BSA and 0.01% NaN_3_) and incubated O/N at RT on rotation. EV-coated beads were then centrifuged at 2,000 xg for 10 min at RT and washed once with BCB prior incubation with primary antibodies [CD71 (Ab08436), CD5L (ab45408) and CD63 (Hybridoma supernatant Clone TEA 3/10.1)] for 30 min at 4°C. After washing with BCB, EV-coated beads were incubated for 30 min at 4°C with secondary antibodies Anti-rabbit Alexa 488 (Invitrogen A11008) or Anti-mouse Alexa488 (Southern Biotec 1032-02). Negative controls included sample-coated beads only incubated with secondary antibodies. Labeled EV-beads were washed twice with BCB before being finally resuspended in PBS and subjected to flow cytometry analysis (FACS Verse, BD). FlowJo software was used to compare median fluorescence intensity (MFI) of EV-coated beads.

### Processing of Human Spleens for Cell Isolation

At the time of organs removal from the donors, whole peripheral blood was removed by perfusion with University of Wisconsin solution (Viaspan). Collected spleens were kept in this solution for their overnight storage at 4°C before processing. Typically, 5–10g of spleen were cut in small pieces (approximately 2 mm^2^) and tissue was disrupted mechanically in the presence of complete Dulbecco’s modified eagle’s medium (DMEM) (Sigma) supplemented with 10% fetal bovine serum (FBS) (Gibco) and 1% penicillin/streptomycin solution (Gibco). Tissue suspension was passed twice through a 70 µm cell strainer washing with complete DMEM medium to obtain a spleen single-cell suspension (SCS). The SCS was let sit for 30 min at RT to sediment aggregated particles and released nucleic acids. The supernatant was then collected avoiding the precipitated components. The cleared SCS was passed once more through 70 µm cell strainer immediately before storage, separation of cell populations, red blood cell lysis and/or analysis by flow cytometry. Viability of cells was assessed by trypan blue (Sigma) exclusion. Total spleen cells were either frozen in freezing solution [FBS supplemented with 10% dimethyl sulfoxide (Sigma)] in liquid nitrogen until use, or used immediately for cell separation and EVs interaction studies. All assays shown were performed with fresh specimens unless stated otherwise.

### Separation of Spleen Cell Populations

Human SCSs were processed to separate T lymphocytes, myeloid cells and RBCs. First, cell suspensions were diluted up to 5 × 10^7^ cells/mL with complete RPMI before layering over Ficoll-Histopaque 1077 (Sigma) to isolate spleen mononuclear cells by density centrifugation. Two-thirds of the splenocytes suspensions were processed by conventional Ficoll density centrifugation and one-third was depleted of T lymphocytes using RosetteSep™ CD3 Depletion Cocktail (Stem cell Technologies) combined to Ficoll density centrifugation following manufacturer’s instructions. After centrifugation, whole mononuclear cells and T lymphocyte-depleted mononuclear cells were collected from the interphase and washed separately with complete RPMI medium. T cells were isolated from 1 × 10^8^ whole mononuclear cells by negative selection with Pan-T Cell Isolation Kit (Miltenyi Biotec) or with CD3+ positive selection magnetic beads (Miltenyi Biotec). T lymphocyte-depleted mononuclear cells were pooled with the cells obtained from the CD3 negative fraction when CD3 MACS separation was performed. T lymphocyte-depleted mononuclear cells were enriched in DCs using EasySep™ Pan-DC Pre-Enrichment Kit (Stem cell Technologies). RBCs pelleted after the conventional Ficoll density centrifugation were collected and washed with complete RPMI. RBCs were depleted from any remaining CD45+ cells with CD45 magnetic beads (Miltenyi Biotec). Counting of viable cells was assessed throughout the whole process by trypan blue (Sigma) exclusion. Enriched T-lymphocytes, DCs and mature RBCs were immediately used in EVs interaction assays.

### Multiparameter Flow Cytometry Analysis

SCS were processed both with and without red blood cell lysis by BD Pharm Lyse buffer following manufacturer’s instructions prior to staining. Briefly, SCS and enriched cell populations were washed with PBS before staining with Fixable Viability Stain 575V (BD Biosciences) at 1:1,000 dilution for 15 min at RT. Then, cells were washed with PBS -1% FBS (Gibco) and incubated for 15 min at RT with antibodies against surface markers (**Supplementary Data Sheet 2**) in Cell separation buffer [(1X MACS separation buffer (Miltenyi Biotec) supplemented with 0.5% BSA (Sigma)]. Cells were further washed with Cell separation buffer prior acquisition in LSR Fortessa flow cytometer (BD). Instrument settings are shown in **Supplementary Data Sheet 3**. For each sample, a minimum of 10^5^ cells was acquired. Controls included unstained samples and single fluorochrome compensation beads. Results were analyzed using FlowJo software 10.6.2.

### Extracellular Vesicles Fluorescent Labeling

Pools of plasma-derived EVs from 10 P*. vivax* patients (*Pv*EVs) and from 10 healthy donors (*h*EVs) were labelled with PKH67 or PKH26 labeling mini kit (Sigma). EV staining was conducted as following our own standard methodology. Briefly, up to 50 µg of EVs diluted up to 1mL with Diluent C were gently mixed with 4 μL of dye in 1 mL of Diluent C and incubated for 5 min at RT. Labeled EVs were then washed 5 times using Amicon^®^ Ultra-15 100-kDa filters units (Millipore) to remove the excess of dye. The washes were performed as follows: samples were centrifuged at 4,000 xg for 10 min, washed twice with 1 mL of 1X PBS and washed three times more with 100 μL of 1X PBS. As a control, 1X PBS was labeled with the fluorescent probes and washed in the same manner as EVs. Protein concentration of labeled-EVs was quantified by BCA assay (Thermo Scientific). Labeled-PBS control was diluted in an equivalent manner.

### Interaction Assays of *Pv*EVs and *h*EVs With Human Splenocytes

Total spleen cells and enriched spleen cell populations were seeded in 24-well plate at 1×10^6^ cells/well and in 96-well plates at 5 × 10^5^ cells/well, respectively, using complete DMEM medium (Sigma) supplemented with EV-depleted 10% FBS (Gibco) and 50 U/mL penicillin–50 µg/mL streptomycin (Gibco). EV-depleted medium was prepared by ultracentrifugation at 100,000 xg for 16h at 4°C. Three microgram per milliliter of protein of PKH26 or PKH67-labelled *Pv*EVs or *h*EVs were added and incubated at 37°C for 3h. In parallel, labeled-PBS was incubated with the cells as a staining background control. After 3h-incubation, total spleen cells and enriched populations were washed in PBS before staining and analyzed by flow cytometry as described above. It is important to mention that due to the ethical and technical restrictions in obtaining human spleen tissue and plasma of *P. vivax* patients from endemic regions, we could only asses EVs-interactions with purified spleens cells from two transplantation donors.

### Confocal Microscopy

Three microgram per milliliter of PKH67 stained *Pv*EVs and *h*EVs were incubated with 0.5 x 10^6^ RBCs in 200 µL of DMEM supplemented with 10% EVs-depleted FBS using µ-Slide 8 Well (Ibidi) at 37°C, 5% CO_2_ for 3 h. Cells were washed at 400 xg for 5 min and resuspended with 200 µL of incomplete DMEM. Confocal images were acquired on a Zeiss LSM 710 Confocal Module coupled to the Zeiss Axio Observer Z1 microscope with a 20x/NA0.40 immersion objective. The intensity of all the channels was standardized through all the experiments. Images of ten different fields were randomly captured and Fiji (ImageJ distribution) software was used for processing images. Total and fluorescent cells were counted manually and percentage of fluorescence cells per field were compared between the conditions (*n* = 10).

### Statistical Analysis

All statistical tests were performed using GraphPad Prism version 8 (GraphPad Software, CA, USA). Statistical significance was determined using Student’s *t*-test. *P*-values <0.05 were considered significant. Comparisons of RBCs-EVs interaction by confocal microscopy was performed using the non-parametric, unpaired and two-sided Mann-Whitney test to calculate p-values. *P*-values <0.05 were considered significant.

## Results

### Immunophenotyping of Human Splenocytes

**Figure 1:**
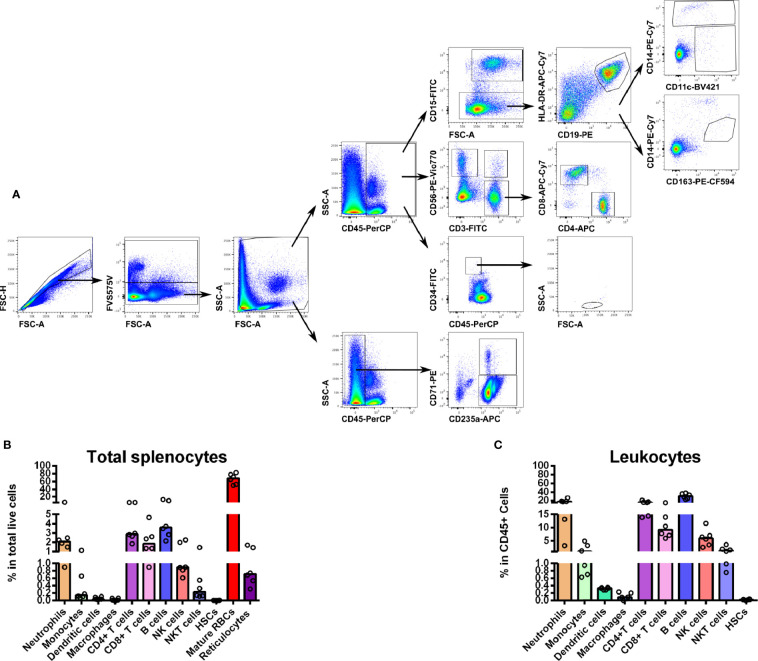
**(A)** Hierarchical multiparameter flow cytometry gate strategy for human spleen cells immunophenotyping. Total spleen cells obtained in suspension after mechanical tissue disruption were assessed for viability and phenotyped using surface markers for several populations. First, singlets were selected by gating events in the diagonal of FSC-H vs. FSC-A plots. Live cells were gated out from the positively stained population with FVS575V viability marker. This total live cell population was then divided in CD45^+^ and CD45^-^ to differentiate whole leukocytes and erythroid cells, respectively. Reticulocytes (CD235a^+^CD71^+^) and mature RBCs (CD235a^+^CD71^-^) were gated in CD45^-^ cells. From the CD45^+^ population we gated neutrophils (CD15^+^), B cells (CD15^-^CD19^+^HLA-DR^+^), monocytes (CD15^-^CD19^-^HLA-DR^+/-^CD14^+^CD11c^+/-^), total DCs (CD15^-^CD19^-^HLA-DR^+/-^CD14^-^ CD11c^+^), macrophages (CD15^-^CD19^-^HLA-DR^+/-^CD14^med^ CD163^+^), T cells (CD3^+^ CD56^-^), NK cells (CD3^-^ CD56^+^), NKT cells (CD3^+^CD56^+^), CD4^+^ T cells (CD3^+^CD56^-^CD4^+^ CD8^-^) and CD8^+^ T cells (CD3^+^CD56^-^CD4^-^CD8^+^). We gated hematopoietic stem cells as a CD45^med^CD34^+^population with a low SSC and medium FSC. **(B)** Distribution of total spleen cells. Frequencies of the different spleen populations, defined as described above were quantified. **(C)** Distribution of total spleen leukocytes. Plots represent the median of six different human spleens analyzed independently.

To gather understanding on the diversity of cell populations that form the human spleen, we performed a multiparameter flow cytometry approach to immunophenotype splenocytes obtained from organ transplantation donors. The methodology involved mechanical tissue disruption followed by exhaustive filtration of the spleen tissue. Importantly, we refrained from using enzymatic digestions in order to avoid alterations of cell surface markers necessary for cell phenotyping. By using a combination of antibodies (**Supplementary Data Sheet 2**) and the gating strategy shown in **Figure 1A**, we characterized the main leukocyte populations as well as mature and immature RBCs (**Figure 1B**). As expected, even after perfusion, erythrocytes (CD45^-^CD235a^+^CD71^-^) comprise the majority of cells in the human spleen accounting for approximately 70% of total cells (**Figure 1B**). Noticeably, we consistently detected a minor population (0.7%) of reticulocytes (CD45^-^CD235a^+^CD71^+^) (**Figure 1B**). Regarding human spleen leukocyte populations (CD45^+^), B cells (CD45^+^CD19^+^) were the most abundant (30%) while CD4^+^ T cells (CD45^+^CD56^-^CD3^+^CD4^+^) and CD8^+^ T cells (CD45^+^CD56^-^CD3^+^CD8^+^) accounted for 16 and 10%, respectively. Innate lymphocytes such as NK cells (CD45^+^CD3^-^CD56^+^) and NKT cells (CD45^+^CD3^+^CD56^+^) were also found (6 and 2%, respectively) (**Figure 1C**). Innate cells from the myeloid lineage, including neutrophils (CD45^+^CD15^+^), represented an abundant population in the human spleen (16% of total leukocytes), although a high variability was observed between different donors (**Figure 1C**). Myeloid phagocytic cells like monocytes (CD45^+^CD19^-^CD14^+^CD11c^+^/^-^) and DCs (CD45^+^CD19^-^CD14^-^CD11c^+^) were also present (2 and 0.3%, respectively). Unexpectedly, macrophages defined as (CD45^+^CD19^-^CD14^med^CD163^+^), were scarce (0.1%) (**Figure 1B**). We also estimated the resident population of hematopoietic stem cells (HSCs) (CD45^med^CD34^+^) finding that 0.02% of cells showed the typical HSC cell surface markers and morphology (**Figure 1C**).

### Isolation and Characterization of Extracellular Vesicles From P*lasmodium vivax* Patients and Healthy Donors

In order to demonstrate the value of this integrated approach to study different human spleen cell subpopulations, we isolated EVs from plasma of ten infected *P. vivax* patients and ten healthy donors using the single-step technology of size exclusion chromatography (de Menezes-Neto et al., 2015). We performed bead-based flow cytometry over the different chromatographic fractions to assess the expression of the classical EV marker tetraspanin CD63, the plasma-derived EV marker CD5L and the reticulocyte marker CD71 to identify the EV-enriched SEC fractions. As previously shown (de Menezes-Neto et al., 2015; Gualdrón-López et al., 2018), EVs were efficiently separated from the bulk of soluble plasma proteins as inferred from the low protein concentration in the EV-enriched fractions with the highest MFI signal of the studied molecular markers (**Supplementary Data Sheet 4A**). CD71 is the major component of reticulocyte-derived exosomes (Harding Stahl, 1983; Pan et al., 1985; Díaz-Varela et al., 2018) and it is a reticulocyte-specific receptor for *P. vivax* (Gruszczyk et al., 2018). Importantly we have recently shown by mass spectrometry-based proteomics and molecular profiling that CD71 is a component of circulating EVs from *P. vivax* patients (Toda et al., 2020). Therefore, we used it as a surrogate molecular marker of EVs derived from *P. vivax*-infected reticulocytes for selection of SEC fractions from individual patients (**Supplementary Data Sheet 4A**) and healthy donors (**Supplementary Data Sheet 4B**) to compose a pool of vesicles for spleen cells interaction experiments.

### Interaction of *Pv*EVs and *h*EVs With Total Spleen Cells

Initially, we explored the capacity of total spleen cells to interact *in vitro* with PKH labeled CD71^high^ *Pv*Evs and *h*EVs (**Supplementary Data Sheet 5A–C**). After incubation of total splenocytes with *Pv*EVs and *h*EVs, we compared the proportion of different cell populations positively stained with PKH26 and PKH67 as a measurement of EVs-cells interaction. Except for CD45^+^CD14^-^CD11c^+^ (**Supplementary Data Sheet 5B**) and CD45^-^CD235a^+^CD71^-^ cells (**Supplementary Data Sheet 5C**) which showed a slightly significant higher proportion of PKH^+^ cells interacting with *Pv*EVs, neither T lymphocytes, NK/NKT cells (**Supplementary Data Sheet 5A**), myeloid cells, nor immature RBCs showed statistically significant difference with EVs from infection compared to control EVs.

### Interaction of *Pv*EVs and *h*EVs With Isolated Spleen Cell Populations

**Figure 2:**
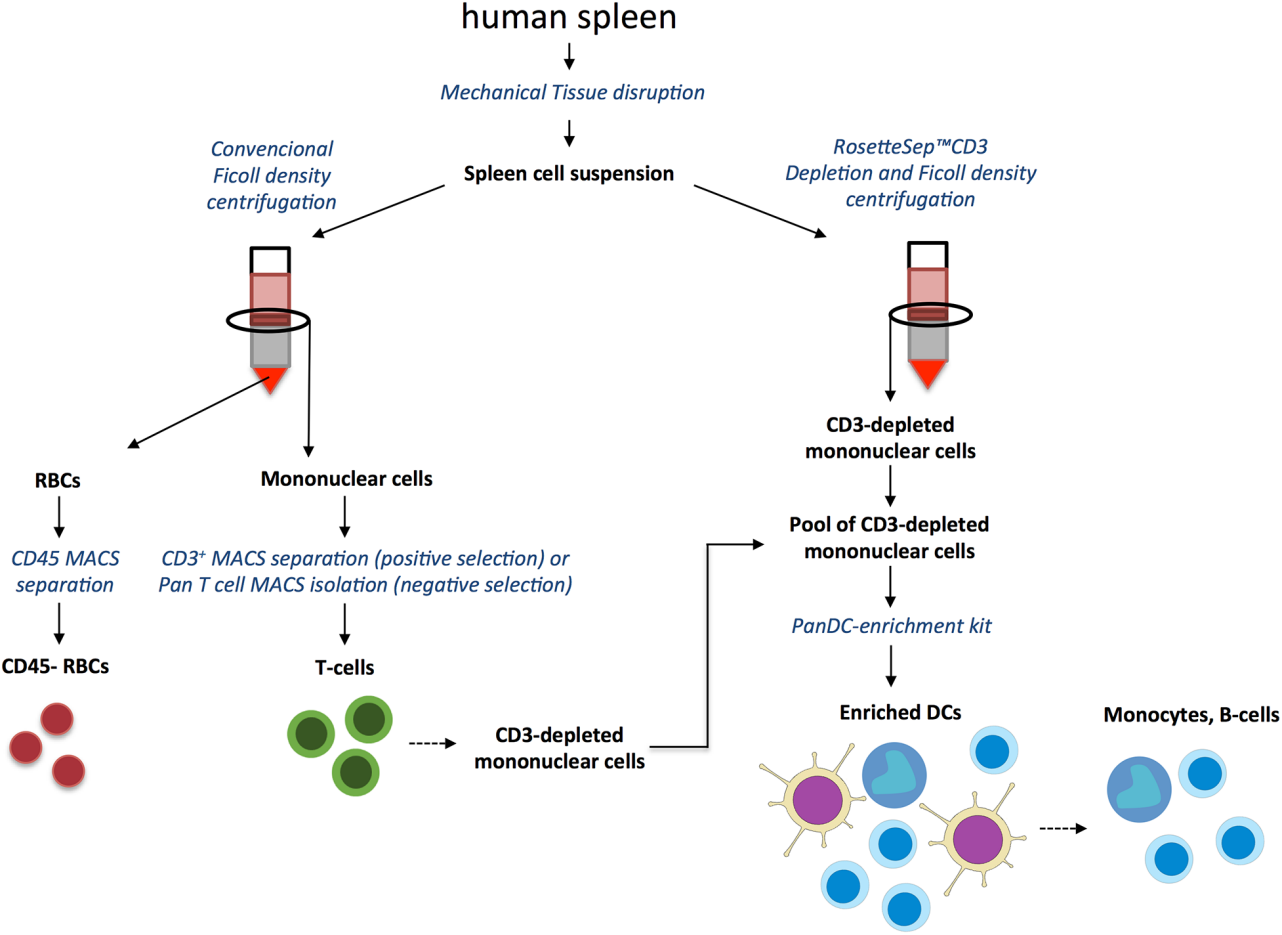
Human spleen T cells, dendritic cells and red blood cells purification strategy. Human spleen was processed to produce a single cell suspension by mechanical tissue disruption and exhaustive filtration. Cell suspensions were processed by conventional Ficoll density centrifugation to isolate mononuclear cells from which T cells were enriched by positive selection with anti-CD3-magnetic beads or by negative selection using Pan-T Cell Isolation Kit. Post-Ficoll sedimented RBCs were further purified by negative selection using anti-CD45 magnetic microbeads to deplete leukocytes. In parallel, DCs were enriched by a first step of T cells depletion using RosetteSep™ CD3 Depletion Cocktail followed by Ficoll density gradient centrifugation. By mixing the CD3- depleted mononuclear fraction obtained by Rossette sep™ and CD3^-^ depleted cells from the flow-through of CD3-positive selection described above we composed a CD3^-^ mononuclear cells pool. DCs were then enriched from the CD3^-^ pool by negative selection using the EasySep™ Pan-DC Pre-Enrichment Kit. The retained fraction rich in monocytes and B cells was also included in the EVs *in vitro* interaction characterization.

**Figure 3:**
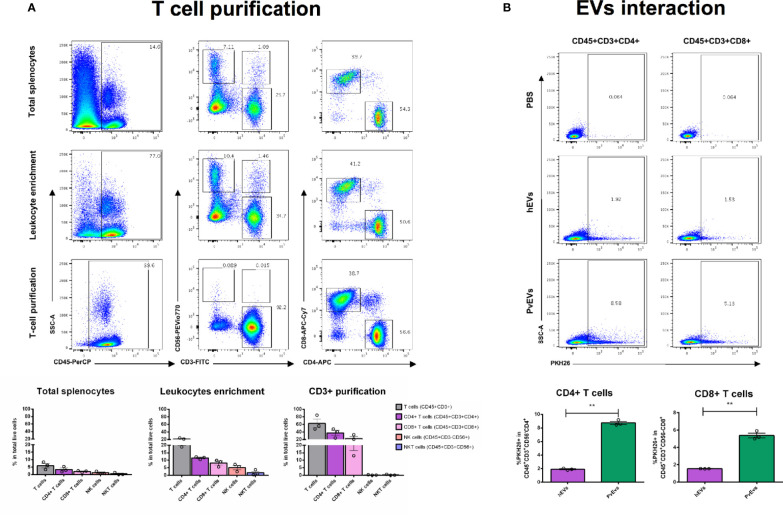
*Pv*EVs and *h*EVs *in vitro* interaction with spleen T cells. **(A)** T cell purification. T cells were purified from spleen cell suspensions by a two-step procedure as shown in **Figure 2**. Cells from all purification steps were stained with fluorescent-conjugated antibodies against surface markers and analyzed by flow cytometry. Representative images show plots of the gating strategy to follow T cell purification. Enrichment quantification shown corresponds to the mean of two purifications from two spleen donors processed independently by negative selection. **(B)** *Pv*EVs and *h*EVs *in vitro* interaction with spleen T cells. Flow cytometry plots showing frequencies of CD4^+^ and CD8^+^ T cells positively labeled with PKH26. PKH26 staining was gated according to cells incubated with PKH26-stained PBS. Frequencies quantification of three technical replicates is shown. Data is representative of two independent experiments performed with two spleen donors. Statistical significance (P<0.05) was assessed using a Student’s *t*-test, **p < 0.001.

In order to address the interaction of *Pv*EVs and *h*EVs with particular human spleen cells of relevance for malaria infections, we have settled a pipeline of cell separation steps involving density centrifugations followed by immunomagnetic cell separation using specific cell surface markers (**Figure 2**) to enrich spleen T-lymphocytes, DCs, and mature RBCs. The rationale for studying EVs interaction with isolated cells is based on the clustered distribution of cells in the human spleen architecture. Therefore, enrichment of immune cells enabled us to assess their interaction with EVs in a more physiological context. Our purification procedure allowed obtaining 70% enrichment of CD3^+^ cells, composed by 56% of CD4^+^ T cells and 37% of CD8^+^ T cells (**Figure 3A**), a proportion previously reported for human spleen T cells (Carpenter et al., 2018b). Similar purification techniques have been previously used achieving 95% pure T cells from peripheral blood (Finney et al., 2004). The differences in purity observed in our experiments could be attributed to the different proportion of T cells in peripheral blood compared to the spleen and the optimization of commercial kits with peripheral blood mononuclear cells (PBMCs) samples and not lymphoid tissue. When enriched T cells were incubated with EVs, we observed a 3-4 fold increased proportion of CD4^+^ T cells and CD8^+^ T cells, respectively, interacting with PKH-labeled *Pv*EVs when compared to *h*EVs (**Figure 3B**). The same result was obtained using positively isolated T cells with anti-CD3 magnetic beads from different spleen donors (**Supplementary Data Sheet 6** and **8A**), suggesting this interaction might be independent of the T cell activation state. Given the T cell suspension contained 30% contaminating cells, we cannot rule out that the increased *Pv*EVs interaction observed with T cells could be due to an indirect effect produced by the response of those contaminant cells to *Pv*EVs.

We conducted interactions assays of the PKH67-labelled *Pv*EVs and *h*EVs with enriched DCs fraction, as well as with monocytes and B cells retained in the DCs-negative fraction of the negative selection step (**Supplementary Data Sheet 7A**). We found that these populations showed interactions above the background level with both types of EVs (**Supplementary Data Sheet 7B**). Interestingly, we found an increased proportion of monocytes (CD45^+^Neutrophils^-^CD19^-^CD14^+^CD11c^+/-^) (1.51 fold) and B cells (CD45^+^ Neutrophils^-^CD19^+^) (2.3 fold) interacting with *Pv*EVs when compared to *h*EVs. This pattern was also observed in the contaminant cells of the DCs enriched fraction (data not shown). Besides the tendency to an increased frequency of DCs (CD45^+^Neutrophils^-^CD19^-^CD14^-^CD11c^+^) interacting with *Pv*EVs when compared to *h*EVs, we could not perform a statistical comparison due to the presence of only two technical replicates due to the low number of cells obtained after the enrichment procedure. Similar results were observed in an independent experiment using a different spleen donor (**Supplementary Data Sheet 8B**).

**Figure 4:**
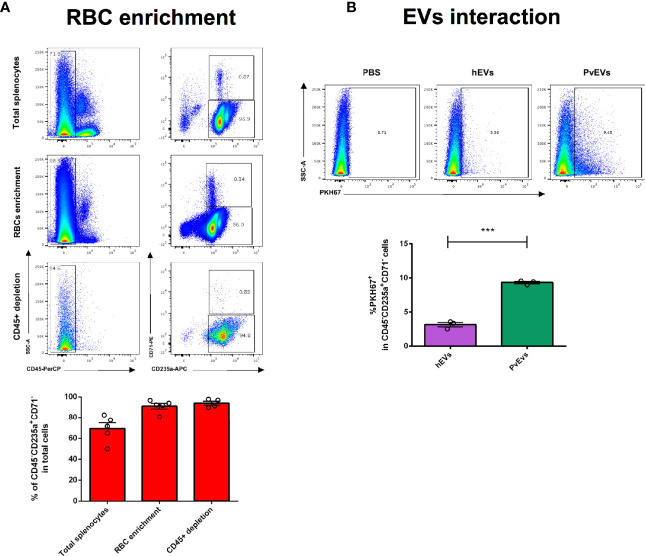
*Pv*EVs and *h*EVs *in vitro* interaction with spleen mature red blood cells by flow cytometry analysis. **(A)** Red blood cells purification. RBCs were purified from spleen cell suspensions by a two-step procedure as shown in **Figure 2**. Enriched RBCs were stained with fluorescent conjugated antibodies against surface markers and analyzed by flow cytometry. Representative images show plots of the RBCs purification procedure. Data shown correspond to the mean and standard deviation of independent purifications from five spleen donors. **(B)** *Pv*EVs and *h*EVs *in vitro* interaction with spleen RBCs cells. Flow cytometry plots showing frequencies of mature RBCs positively stained with PKH67. PKH67 staining was gated according to cells incubated with PKH67-stained PBS. Frequencies quantification of three technical replicates is shown. Data is representative of two independent experiments performed with two spleen donors. Statistical significance (P<0.05) was assessed using a Student’s *t*-test, ***p < 0.0001.

**Figure 5:**
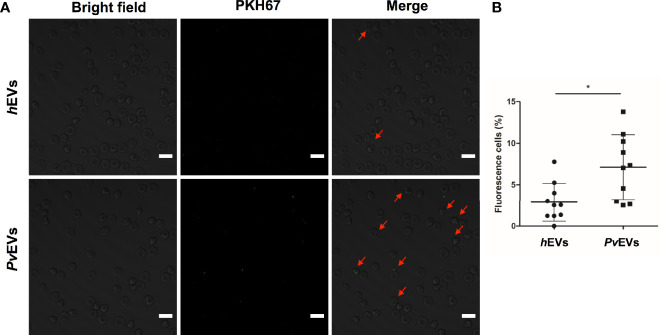
*Pv*EVs and *h*EVs *in vitro* interaction with spleen mature red blood cells by confocal microscopy. **(A)** Purified RBCs were incubated with PKH67-labelled *Pv*EVs and *h*EVs. Confocal microscopy analysis shows RBCs with green fluorescent dots in the membrane (red arrows). Scale bar: 10 µm. **(B)** Quantification of RBCs with positive staining for PKH67. Data represent the mean and standard deviation of the percentage of cells with fluorescent green dots per field (n=10) in each condition. Statistical significance (P<0.05) was assessed using unpaired and two-sided, Mann-Whitney test, *p < 0.05.

In addition, we also explored whether mature spleen RBCs (CD45^-^CD235a^+^CD71^-^) could interact with *Pv*EVs and *h*EVs. To consistently test this interaction, we depleted contaminant CD45^+^ leukocytes in a two-step purification procedure achieving 99% purity of RBCs (**Figure 4A**). Remarkably, *in vitro* interaction assays showed that purified mature spleen RBCs strongly interact with *Pv*EVs as a three-fold higher proportion of cells stained with PKH67-labelled *Pv*EVs were observed when compared to *h*EVs (**Figure 4B**). Interestingly, an increased number of green fluorescent dots was observed in the plasma membrane of spleen RBCs incubated with PKH67-labelled *Pv*EVs as compared with *h*EVs (**Figure 5**). A similar increased interaction of RBC with *Pv*EVs was observed in an independent experiment using a different spleen donor (**Supplementary Data Sheet 8C**).

## Discussion

In this study we have performed an integrated characterization of human spleen cells using multiparameter flow cytometry, a powerful technique that has been systematically applied in immunophenotyping in clinical and research settings (O’Donnell et al., 2013). In our approach, we have employed spleens from transplantation donors as a source of healthy splenic tissue followed by mechanical tissue disruption to prepare viable spleen single cell suspensions with intact cell surface markers. Our work allowed the quantification of several immune cells including neutrophils, monocytes, DCs, T and B lymphocytes, NK and NKT cells as well as resident reticulocytes, HSCs and mature RBCs (**Figure 1**). In spite of the limitations faced in human spleen studies due to ethical and practical constraints, diverse studies of human spleen specific cell populations have provided a great understanding of the function and phenotype of resident splenic immune cells (Colovai et al., 2004; Langeveld et al., 2006; Velásquez-Lopera et al., 2008; Meinderts et al., 2017; Nagelkerke et al., 2018) and stromal cells such as fibroblasts and endothelial cells (Briard et al., 2002; Rosti et al., 2013) under physiological and pathological conditions. However, few studies applied a comprehensive approach that addresses the cellular diversity of the spleen, taking into account not only leukocytes and stromal cells, but also immature and mature RBCs. The immunophenotyping performed in this work demonstrated that the frequencies of T cells, B cells, monocytes and neutrophils in the spleen are in agreement with a previous report where a similar holistic approach was conducted to quantify leukocytes from the spleen and other human lymphoid organs across several clinical conditions (Carpenter et al., 2018a). Innate lymphoid cells such as NK and NKT cells showed similar proportions to those previously reported in the human spleen (Colovai et al., 2004; Langeveld et al., 2006). Prior studies have shown that macrophages and DCs in the human spleen represent a 9 and 0.7% of mononuclear cells, respectively (Mcilroy et al., 2001). The reduced amount of these two populations in our study is likely due to the absence of collagenase treatment during tissue disaggregation, which might be necessary to release these cells from parenchyma. As expected, mature RBCs account for the great majority of splenocytes reflecting the primordial function of this organ in blood filtration. Our immunophenotyping also revealed a small proportion of young CD71^+^ reticulocytes, classically believed to be restricted to the human bone marrow. Importantly, CD71^+^ reticulocytes are the host cell of *P. vivax* (Kitchen, 1937), and our previous studies have indicated that *P. vivax* parasite can be found in the spleen in human infections (Machado Siqueira et al., 2012; Elizalde-Torrent et al., 2018). Of note, spleens in this study were removed during transplantation surgery after whole-body perfusion. This implies that spleen tissue used for our experiments is largely depleted from circulating cells. Whether the presence of these reticulocytes is used by the parasite to establish spleen infections remains to be determined.

Given the importance of the spleen in parasite removal and immune response during malaria (Engwerda et al., 2005; del Portillo et al., 2012) and the role of secreted extracellular vesicles in cell communication processes involved in pathogenesis of this infectious disease (Babatunde et al., 2020), we explored the *in vitro* interaction of circulating EVs from *P. vivax* patients with total spleen cells by a flow cytometry approach. Overall, our results showed that with the exception of DCs and mature RBCs, no other cell populations differentially interacted with *Pv*EVs as compared to *h*EVs (**Supplementary Data Sheet 2**). Despite the statistical significance of this result, the low number of PKH67 positively stained cells in all populations studied may be a limitation in the interpretation of these interactions. However, the increased interaction observed in DCs when whole splenocytes were in contact with PKH-labeled *Pv*EVs can reflect its specific phagocytosis. Indeed, we have previously demonstrated that human reticulocyte-derived exosomes are specifically taken up by DCs in a Siglec-1-dependent manner (Díaz-Varela et al., 2018) and circulating EVs in patients with acute *P. vivax* infection contain parasite proteins (Toda et al., 2020). In the absence of supportive evidence, this remains to be determined.

A clear limitation of the total splenocytes-EVs approach in delineating physiological relevant interactions is the impossibility of recapitulating the structural features derived from the spleen microcirculation (Mebius and Kraal, 2005; Ferrer et al., 2014). We expect that blood circulating EVs are restricted to encounter parenchymal and immune cells enriched in particular spleen compartments. Such site-specific interactions with the distinct spleen cell populations could trigger differential physiological responses and alter local signaling at autocrine and paracrine levels. In order to overcome this limitation, we explored *Pv*EVs interactions with enriched human spleen cell populations of physiological relevance for malaria immune response and mechanism of anemia, two processes in which the spleen is a major player (del Portillo et al., 2012). We designed a multistep cell purification methodology to enrich T cells, DCs and mature RBCs using density centrifugation and sequential magnetic immunocapture (**Figure 2**). Our results showed that T cells enrichment, both, by negative (**Figure 3A**) and positive selection (**Supplementary Data Sheet 6A**) resulted in around 56–70% pure and viable CD45^+^CD3^+^ cells indicating effective cell separation. The enrichment method for DCs, however, showed limitations since a very low yield and compromised purity was observed (**Supplementary Data Sheet 7A**). Notably, the DC enrichment commercial kit used has been standardized using PBMCs in which the proportion of leukocytes differs from lymphoid tissue such as the splenic one. We can attribute the high contamination of B cells in our DC enriched sample to this fact. In contrast, the method for RBCs purification was highly efficient at the initial density centrifugation step as we managed to obtain 99% pure RBCs. Importantly, the subsequent CD45^+^ cells depletion allowed to eliminate the totality of contaminating leukocytes (**Figure 4A**).

Following this spleen-cell population separation approach we demonstrated an increased interaction of monocytes, B cells and T cells from the human spleen with circulating EVs from *P. vivax* infected patients compared to EVs from healthy donors (**Supplementary Data Sheet 7B** and **Figure 3B**). EVs are now well-known mediators of the regulation of immune responses under physiological and pathological conditions such as cancer and infection (Robbins and Morelli, 2014). Given that the spleen is a key organ to generate immune responses, components potentially present on EVs during *P. vivax* infection (e.g., parasite antigens) could contribute to the initiation and/or the promotion of such responses. Indeed, it is widely demonstrated that EVs derived from malaria infected cells contain parasite proteins (Martin-Jaular et al., 2011; Mantel et al., 2013; Antwi-Baffour et al., 2016) and that they can induce proinflammatory responses in innate immune cells such as macrophages, neutrophils and monocytes (Couper et al., 2010; Mantel et al., 2013; Sisquella et al., 2017). One potential mechanism for the *Pv*EVs increased interaction with splenic phagocytic cells (monocytes and B cells) could be the recognition by Fc receptors of immune complexes formed by host antibodies and parasite antigens in EVs. Indeed, VIR proteins are associated to *Pv*EVs (Toda et al., 2020) and anti-VIR IgGs have been detected in the serum of *P. vivax* patients (Oliveira et al., 2006). Although there are no published reports that have demonstrated the formation of immune complexes with EVs from malaria patients, EV-derived circulating immune complexes have been described in chronic Chagas disease (Díaz Lozano et al., 2017), reinforcing this hypothetical interaction mechanism.

We previously demonstrated that immunization with exosomes derived from reticulocytes infected with *P. yoelii* 17X, a rodent malaria strain that preferentially invades reticulocytes and often used as a model for *P. vivax* malaria, elicited humoral responses (Martin-Jaular et al., 2011) and induced non-exhausted effector memory T cells that conferred a spleen-dependent long-lasting protection (Martín-Jaular et al., 2016). The induction of efficient T cell responses by EVs is one of the most extensively studied EV-mediated immune mechanisms (Zitvogel et al., 1998; Théry et al., 2002a; Segura et al., 2005; Menningher et al., 2020). Its relevance for immunointerventions was initially investigated in cancer (Wolfers et al., 2001) and later in models of infection (Aline et al., 2004; Beauvillain et al., 2007; Schnitzer et al., 2010; Cheng and Schorey, 2013). T cell responses are also relevant for the control of *P. vivax* infection, where a protective role of CD8^+^ T cells against blood-stage *P*. vivax parasites has been suggested (Burel et al., 2016; Junqueira et al., 2018). Importantly, HLA class I molecules have been identified in human reticulocyte-derived exosomes (Díaz-Varela et al., 2018).

One of the homeostatic functions of the spleen is the destruction of senescent and damaged RBCs in a process called erythrophagocytosis. This is mediated by red pulp macrophages and neutrophils and promoted by IgG opsonization (Meinderts et al., 2017). Interestingly, we observed a three-fold increase of spleen isolated RBCs interacting with *Pv*EVs compared with its *h*EVs counterparts (**Figure 4B**). In addition, confocal microscopy showed that this interaction occurs at the membrane of RBCs (**Figure 5**). This observation can have important implications for the pathophysiological mechanism of anemia in malaria. EVs derived from malaria infected RBCs that contain parasite proteins and physically interact with mature healthy RBCs in the spleen, could induce its opsonization and destruction by phagocytes leading to severe anemia. A similar mechanism has been proved for *Trypanosoma brucei* secreted EVs in the induction of anemia in sleeping sickness patients (Szempruch et al., 2016). If such EV-mediated opsonization of healthy RBCs is involved in severe anemia in vivax malaria needs further investigation.

In summary, to the best of our knowledge, our data show the first integrated analysis of the human spleen at cellular level including HSCs, lymphoid, myeloid and erythroid lineages. We implemented a methodology for the sequential enrichment of specific human spleen cell populations to study their *in vitro* interaction with plasma-derived EVs from *P. vivax* infected patients. These studies show that monocytes, T and B lymphocytes as well as mature RBCs interact with circulating EVs from patients. Further exploration of the functional relevance of such interactions can contribute to not only our understanding of important pathophysiological processes involving the spleen in vivax malaria, but also to rational vaccine development.

## Data Availability Statement

The raw data supporting the conclusions of this article will be made available by the authors, without undue reservation.

## Ethics Statement

The studies involving human participants were reviewed and approved. 1. Plasma samples from *P. vivax*-infected patients (PV) were collected at the Hospital of the Fundação de Medicina Tropical Doutor Heitor Vieira Dourado (Manaus, Amazonas, Brazil). 2. Plasma from healthy donors (HD) was obtained at the Hospital Germans Trias i Pujol (Badalona, Barcelona, Spain) after expressed consent from the donors. 3. Human spleen donations for biomedical research received written consent from family members and was in accordance with the protocol approved by the Ethics Committee for Clinical Research of the Hospital Germans Trias i Pujol. The patients/participants provided their written informed consent to participate in this study.

## Author Contributions

MG-L, MD-V, HT, IA-H, and LP-C performed experiments of spleen immunophenotyping and cell purification. MG-L, MD-V, and HT performed experiments EVs purification and EVs–splenocytes interaction. MG-L, MD-V, and MF-S analyzed the data. MG-L, MD-V, CF-B, and HP suggested the experiments. RL and ML contributed materials. MG-L, MD-V, and HP drafted the manuscript. HP conceived this study. All authors contributed to the article and approved the submitted version.

## Funding

MG-L is a postdoctoral fellow supported by the Plan Estratégico de Investigación e Innovación en Salud (PERIS, SLT002/16/00179) of the Generalitat de Catalunya, Spain. MD-V and HT are predoctoral fellows supported by Secretaria d’Universitats i Recerca del Departament d’Economia i Creixement, Generalitat de Catalunya (2017 FI_B2_00029) and (2017FI_B1_00202). IA-H is a predoctoral fellow supported by the Ministerio de Economia y Competitividad (FPI BES-2017081657). We acknowledge the support from the Spanish Ministry of Science, Innovation and Universities, “Centro de Excelencia Severo Ochoa 2013-2017”, SEV-2012-0208, and “Secretaria d’Universitats i Recerca del Departament d’Economia i Coneixement de la Generalitat de Catalunya” (2017SGR595). This research is part of ISGlobal’s Program on the Molecular Mechanisms of Malaria, which is partially supported by the Fundación Ramón Areces. Work in the laboratory of CF-B and HP is funded by the Ministerio Español de Ciencia e Innovación (PID2019-111795RB-I00) and by the Network of Excellency in Research and Innovation on Exosomes (REDiEX) (SAF2015-71231-REDT). ISGlobal and IGTP are members of the CERCA Programme, Generalitat de Catalunya.

## Conflict of Interest

The authors declare that the research was conducted in the absence of any commercial or financial relationships that could be construed as a potential conflict of interest.

## Supplementary Material

Supplementary Data Sheet 1 Summary of spleen tissue processed for immunophenotyping and EVs interaction experiments.

Supplementary Data Sheet 2 Fluorescent conjugated antibodies used for immunophenotyping of spleen cells.

Supplementary Data Sheet 3 LSR Fortessa flow cytometer (BD) configuration indicating laser intensities and acquisition channels.

Supplementary Data Sheet 4 Molecular characterization of plasma-derived EVs from healthy donors and *P. vivax* infected patients purified by size exclusion chromatography (SEC) and analyzed by flow cytometry bead-based assay. **(A)** Distribution of CD5L, CD71 and CD63 markers in SEC fractions from plasma of *P. vivax* patients. Negative Control (Cont) refers to a mix of all fractions incubated with anti-rabbit Alexa 488- 2^ary^ antibodies. Dashed rectangles refer to fractions selected to constitute EVs pools for PKH staining and *in vitro* interaction studies. **(B)** Distribution of CD5L and CD71 EVs markers in the SEC fractions from plasma of healthy donors. Negative Control (Cont) refers to Fractions F8 incubated with anti-rabbit Alexa 488- 2^ary^ antibodies.

Supplementary Data Sheet 5 *In vitro* interaction of *Pv*EVs and *h*EVs with total human spleen cells. Total spleen cells were obtained and frozen as described in the Methods section. Splenocytes were thawed and incubated with PKH26 labelled *Pv*EVs, *h*EVs and PBS for 3h. Cells were washed and then stained with surface markers. Positive PKH26 staining was gated according to unlabeled cells. **(A)** Flow cytometry plots showing frequencies of NK, NKT, total T lymphocytes, CD4^+^ T cells and CD8^+^ T cells positive for PKH26. Frequencies quantification of three technical replicates is shown. **(B)** Flow cytometry plots of spleen monocytes and DCs showing frequencies of positive PKH26 cells. Frequencies quantification of three technical replicates is shown. **(C)** Flow cytometry plots of spleen reticulocytes and mature RBC showing frequencies of positive PKH26 cells. Frequencies quantification of three technical replicates is shown. Data shows a representative experiment of two different spleen donors analyzed independently. Statistical significance (P<0.05) was assessed using a Student’s *t*-test, **p<0.05*.

Supplementary Data Sheet 6 *Pv*EVs and *h*EVs *in vitro* interaction with spleen T cells isolated by positive selection. **(A)** T cell purification. T cells were purified from spleen cell suspensions by a two-step procedure involving density gradient centrifugation and CD3^+^ immunomagnetic cell isolation. Cells from all purification steps were stained with fluorescent-conjugated antibodies against CD4 and CD8 surface markers and analyzed by flow cytometry. Plot corresponds to a representative image showing T cell enrichment quantification of two independent experiments. **(B)** *Pv*EVs and *h*EVs *in vitro* interaction with spleen T cells. Frequencies of CD4^+^ and CD8^+^ T cells positively labeled with PKH26. PKH26 staining was gated according to cells incubated with PKH26-stained PBS. Frequencies quantification of three technical replicates is shown. Data is representative of two independent experiments performed with two spleen donors. Statistical significance (P<0.05) was assessed using a Student’s *t*-test, ** p<0.05*.

Supplementary Data Sheet 7 *Pv*EVs and *h*EVs *in vitro* interaction with spleen phagocytic cells. **(A)** Dendritic cells enrichment. DCs were enriched from spleen cell suspensions by a two-step procedure as shown in **Figure 2**. Enriched cells were stained with fluorescent-conjugated antibodies against surface markers and analyzed by flow cytometry. Images show flow cytometry plots of the gating strategy to follow DC enrichment through the purification procedure. Enrichment quantification shown corresponds to one purification from one spleen donor. **(B)** *Pv*EVs and *h*EVs *in vitro* interaction with spleen phagocytic cells. Flow cytometry plots showing frequencies of DCs, monocytes and B cells positively labeled with PKH67. PKH67 staining was gated according to cells incubated with PKH67-stained PBS. Frequencies quantification of three technical replicates is shown. Data represents mean and standard deviation of technical replicates of one experiment performed with one spleen donor. Statistical significance (P<0.05) was assessed using a Student’s t-test, *p<0,001.

Supplementary Data Sheet 8 Summary of *Pv*EVs and *h*EVs *in vitro* interaction with spleen cells from biological sample 2. **(A)** *Pv*EVs and *h*EVs *in vitro* interaction with spleen T cells isolated by positive selection. T cells were purified from spleen cell suspensions by a two-step procedure involving density gradient centrifugation and CD3^+^ immunomagnetic cell isolation. Enriched cells were tested for its *in vitro* interaction with PKH67 labeled *Pv*EVs and *h*EVs. Plots show frequencies of CD4^+^ and CD8^+^ T cells positively labeled with PKH26. **(B)** *Pv*EVs and *h*EVs *in vitro* interaction with spleen phagocytic cells. Phagocytic cells were enriched by depletion of CD3^+^ cells. Cells were tested for its *in vitro* interaction with PKH67 labeled *Pv*EVs and *h*EVs. Plots shows frequencies of DCs (CD45^+^Neutrophils^-^CD19^-^CD14^-^CD11c^+^), Monocytes (CD45^+^ Neutrophils^-^CD19^-^CD14^+^CD11c^-^) and B cells (CD45^+^ Neutrophils^-^CD19^-^) positively stained with PKH67. **(C)** *Pv*EVs and *h*EVs *in vitro* interaction with spleen mature red blood cells (RBCs). RBCs were purified from spleen cell suspensions by a two-step procedure as shown in **Figure 2**. Enriched cells were tested for its *in vitro* interaction with PKH26 labeled *Pv*EVs and *h*EVs. Plots shows frequencies of mature RBCs (CD45^-^CD71^-^) positively stained with PKH67. PKH67 was gated according to cells incubated with PKH67-stained PBS. All plots shows frequencies of three technical replicates. Statistical significance (P<0.05) was assessed using a Student’s *t*-test, **p<0,01, **p<0,001*.
