# Supplementary material for: Multiparameter Flow Cytometry Analysis of the Human Spleen Applied to Studies of Plasma-Derived EVs From Plasmodium vivax Patients
Source: Front Cell Infect Microbiol. 2021 Mar 1;11:596104. doi: 10.3389/fcimb.2021.596104 (PMC7957050; doi:10.3389/fcimb.2021.596104)
Supplement: Supplementary Data Sheet 1 — Summary of spleen tissue processed for immunophenotyping and EVs interaction experiments. [file DataSheet_1.pdf]

Tissue reference name	State of cells	Experiment	Age donor	Sex donor	Clinical information (cause of death)
100816	Frozen	Total splenocytes Evs interaction	59	Male	Cardio-respiratory arrest and cerebral anoxia
140717	Fresh	Cell separation Sample 1 and EVs interaction assays	52	Male	Ischemic stroke
210917	Fresh	Cell separation Sample 2 and EVs interaction assays	25	Female	Cardio-respiratory arrest and cerebral anoxia
20181114	Fresh	Immunophenotyping, T cell isolation by negative selection and EVs interaction assays 1	61	Female	Hemorrhagic stroke
20181204	Fresh	Immunophenotyping, T cell isolation by negative selection and EVs interaction assays 2	53	Male	Cardio-respiratory arrest and cerebral anoxia
20190201-01	Fresh	Immunophenotyping	N/T	N/T	N/T
20190201-02	Fresh	Immunophenotyping	65	Male	N/T
20190207	Fresh	Immunophenotyping	62	Female	Hemorrhagic stroke
20190222	Fresh	Immunophenotyping	66	Male	Cardio-respiratory arrest and cerebral anoxia
