# Supplementary material for: Multiparameter Flow Cytometry Analysis of the Human Spleen Applied to Studies of Plasma-Derived EVs From Plasmodium vivax Patients
Source: Front Cell Infect Microbiol. 2021 Mar 1;11:596104. doi: 10.3389/fcimb.2021.596104 (PMC7957050; doi:10.3389/fcimb.2021.596104)
Supplement: Supplementary Data Sheet 2 — Fluorescent conjugated antibodies used for immunophenotyping of spleen cells. [file DataSheet_2.pdf]

Panel for RBCs

antigen	*fluorochrome*	*clone*	*manufacturer*	*reference*	*dilution*
CD45	PerCP	5B1	Miltenyi biotec	130-113-120	1/100
		2D1	BD Biosciences	345809	1/50
CD235a	APC	REA175	Miltenyi biotec	130-100-270	1/100
CD71	PE	AC102	Miltenyi biotec	130-091-728	1/400

Panel for T-cells/NK cells

antigen	*fluorochrome*	*clone*	*manufacturer*	*reference*	*dilution*
CD45	PerCP	5B1	Miltenyi biotec	130-113-120	1/100
		2D1	BD Biosciences	345809	1/50
CD3	FITC	REA613	Miltenyi biotec	130-113-138	1/400
		SK7	StemCell Technologies	60127FI.1	1/200
CD8	APC-Cy7	SK1	Biolegend	344714	1/200
			BD Biosciences	641400	1/400
CD4	APC	RPA-T4	Biolegend	300514	1/100
CD56	PE-Vio770	AF12-7H3	Miltenyi Biotec	130-098-132	1/100

Panel for monocytes/DCs/B-cells/neutrophils/macrophages

antigen	*fluorochrome*	*clone*	*manufacturer*	*reference*	*dilution*
CD45	PerCP	5B1	Miltenyi biotec	130-113-120	1/100
		2D1	BD Biosciences	345809	1/50
CD14	PE-Cy7	M5E2	Biolegend	301813	1/100
	BV-785		Biolegend	301839	1/30
CD11c	BV-421	3.9	Biolegend	301627	1/100
	BV-711		Biolegend	301629	1/30
HLA-DR	APC-Cy7	L243	Biolegend	307617	1/100
			BD Biosciences	641393	1/200
CD19	PE	REA675	Miltenyi biotec	130-113-646	1/400
	BV-510	HIB19	Biolegend	302241	1/50
CD15	FITC	VIMC6	Miltenyi biotec	130-114-010	1/400
CD86	APC	REA968	Miltenyi biotec	130-116-264	1/100
CD163	PE-CF594	GHI/61	BD Biosciences	562670	1/200

Panel for hematopoietic stem cells

antigen	*fluorochrome*	*clone*	*manufacturer*	*reference*	*dilution*
CD45	PerCP	5B1	Miltenyi biotec	130-113-120	1/100
		2D1	BD Biosciences	345809	1/50
CD34	FITC	AC136	Miltenyi biotec	130-081-001	1/100
