# Supplementary material for: Multiparameter Flow Cytometry Analysis of the Human Spleen Applied to Studies of Plasma-Derived EVs From Plasmodium vivax Patients
Source: Front Cell Infect Microbiol. 2021 Mar 1;11:596104. doi: 10.3389/fcimb.2021.596104 (PMC7957050; doi:10.3389/fcimb.2021.596104)
Supplement: Supplementary Data Sheet 3 — LSR Fortessa flow cytometer (BD) configuration indicating laser intensities and acquisition channels. [file DataSheet_3.pdf]

VIOLET LASER (405 nm; 100 mW)

PMT POSITION	SPLITTER	BP FILTER	Name	Common Fluorochromes
A	750 LP	780/60	***V780***	***BV786***
B	670 LP	710/50	***V710***	***BV711***
C	630 LP	660/20	***V660***	***BV650***
D	595LP	605/40	***V605***	***BV605***
E	550 LP	585/42	***V585***	***BV570***
F	535 LP	560/40	***V560***	***BV570***
G	505 LP	515/20	***V515***	***BV510, V500, AmCyan***
H	NONE	450/50	***V450***	***BV421, PACIFIC BLUE, V450, ALEXA 405***

BLUE LASER (488 nm; 100 mW)

PMT POSITION	SPLITTER	BP FILTER		Common Fluorochromes
B	635LP	695/40	***B695***	***PerCP, PerCP-Cy5.5, 7-AAD, PI***
E	505LP	515/20	***B530***	***FITC, GFP, CFSE, ALEXA 488***
F	NONE	488/10	***SSC***	***-***

GREEN LASER (532 nm; 150 mW)

PMT POSITION	SPLITTER	BP FILTER		Common Fluorochromes
A	750 LP	780/60	***G780***	***PECy7***
B	670 LP	705/70	***G710***	***PECy5.5***
C	635 LP	670/14	***G670***	***PECy5***
D	600 LP	616/23	***G610***	***PECF594, PE-TexasRed, PI***
E	NONE	575/26	***G575***	***PE***

RED LASER (640 nm; 40 mW)

PMT POSITION	SPLITTER	BP FILTER		Common Fluorochromes
A	735 LP	780/60	***R780***	***APCCy7, APCH7***
B	690 LP	710/50	***R710***	***ALEXA 700, APC R-700***
C	NONE	660/20	***R660***	***APC, ALEXA 647***
