# Supplementary figures and images for: Multiparameter Flow Cytometry Analysis of the Human Spleen Applied to Studies of Plasma-Derived EVs From Plasmodium vivax Patients
Source: Front Cell Infect Microbiol. 2021 Mar 1;11:596104. doi: 10.3389/fcimb.2021.596104 (PMC7957050; doi:10.3389/fcimb.2021.596104)

A

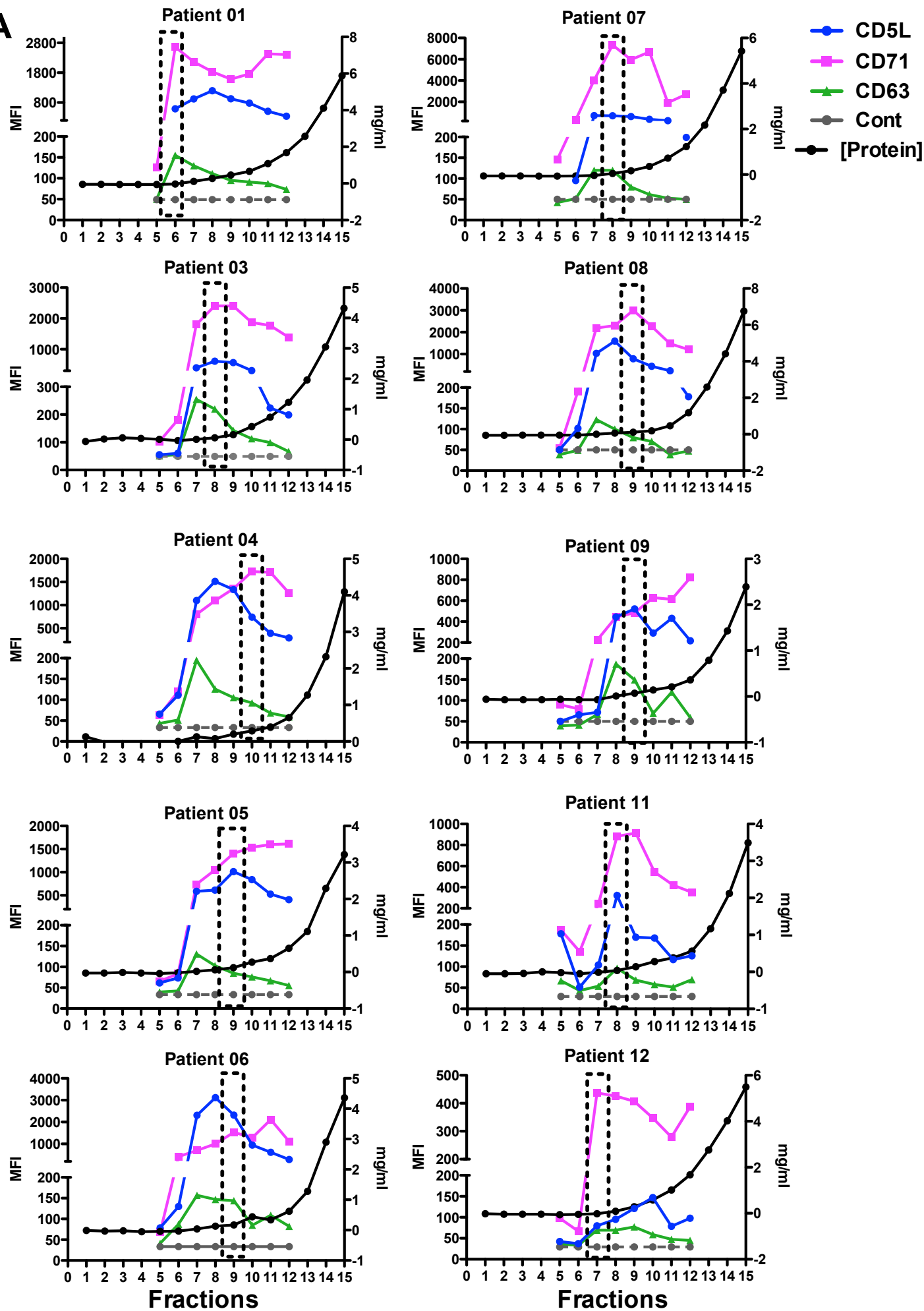

**B**

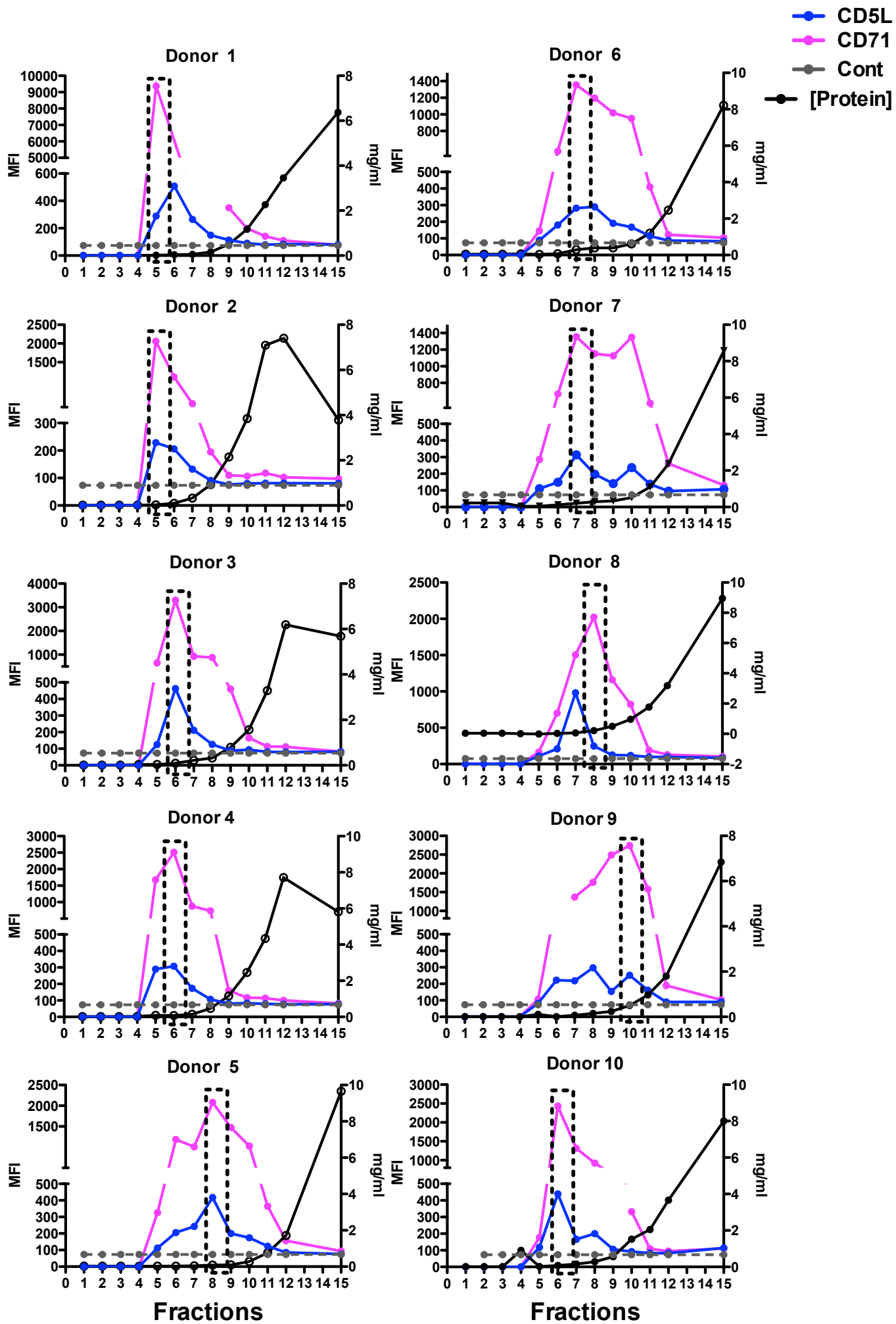

Supplement: Supplementary Data Sheet 4 — Molecular characterization of plasma-derived EVs from healthy donors and P. vivax infected patients purified by size exclusion chromatography (SEC) and analyzed by flow cytometry bead-based assay. (A) Distribution of CD5L, CD71 and CD63 markers in SEC fractions from plasma of P. vivax patients. Negative Control (Cont) refers to a mix of all fractions incubated with anti-rabbit Alexa 488- 2ary antibodies. Dashed rectangles refer to fractions selected to constitute EVs pools for PKH staining and in vitro interaction studies. (B) Distribution of CD5L and CD71 EVs markers in the SEC fractions from plasma of healthy donors. Negative Control (Cont) refers to Fractions F8 incubated with anti-rabbit Alexa 488- 2ary antibodies. [file DataSheet_4.pdf]

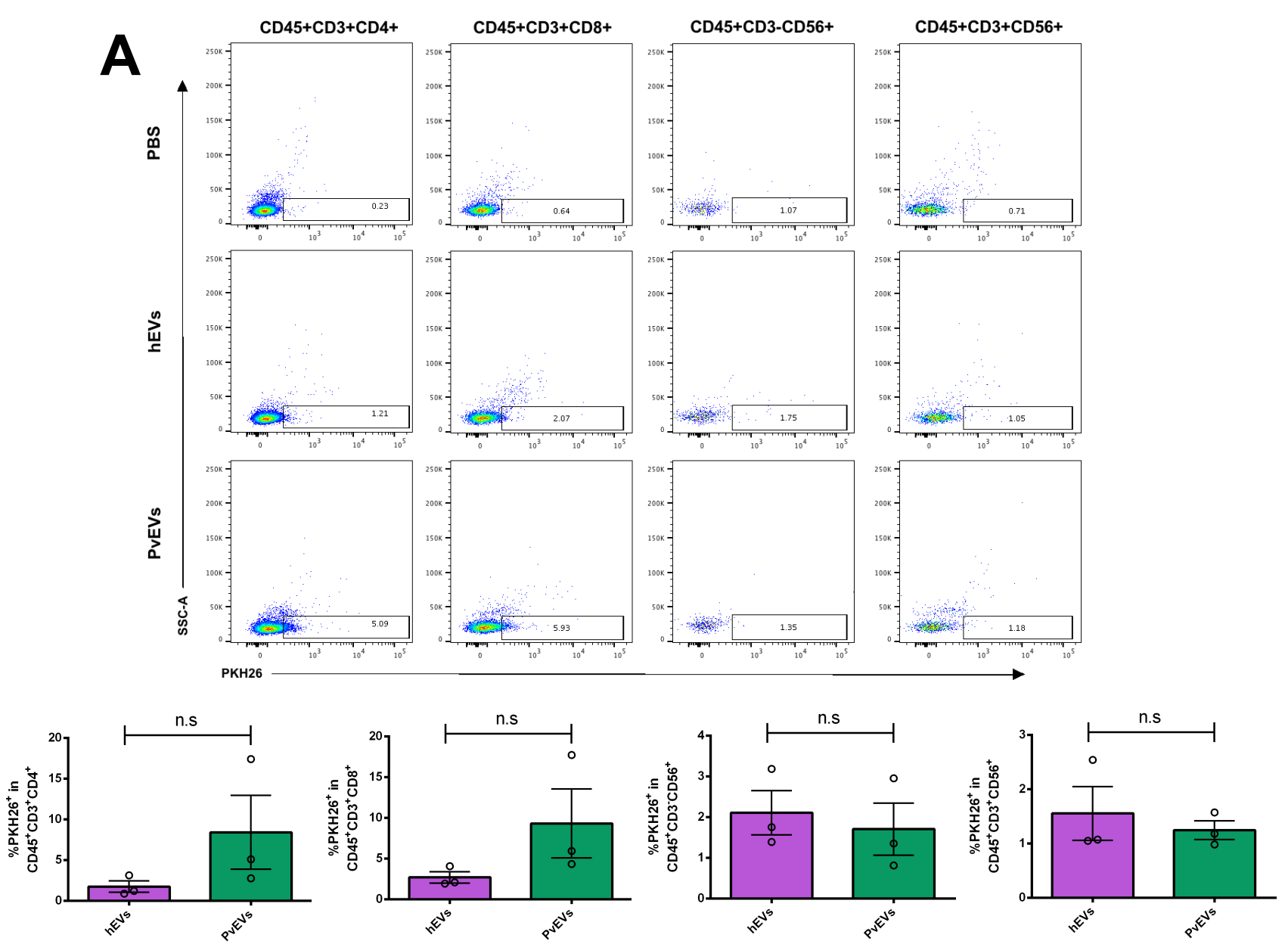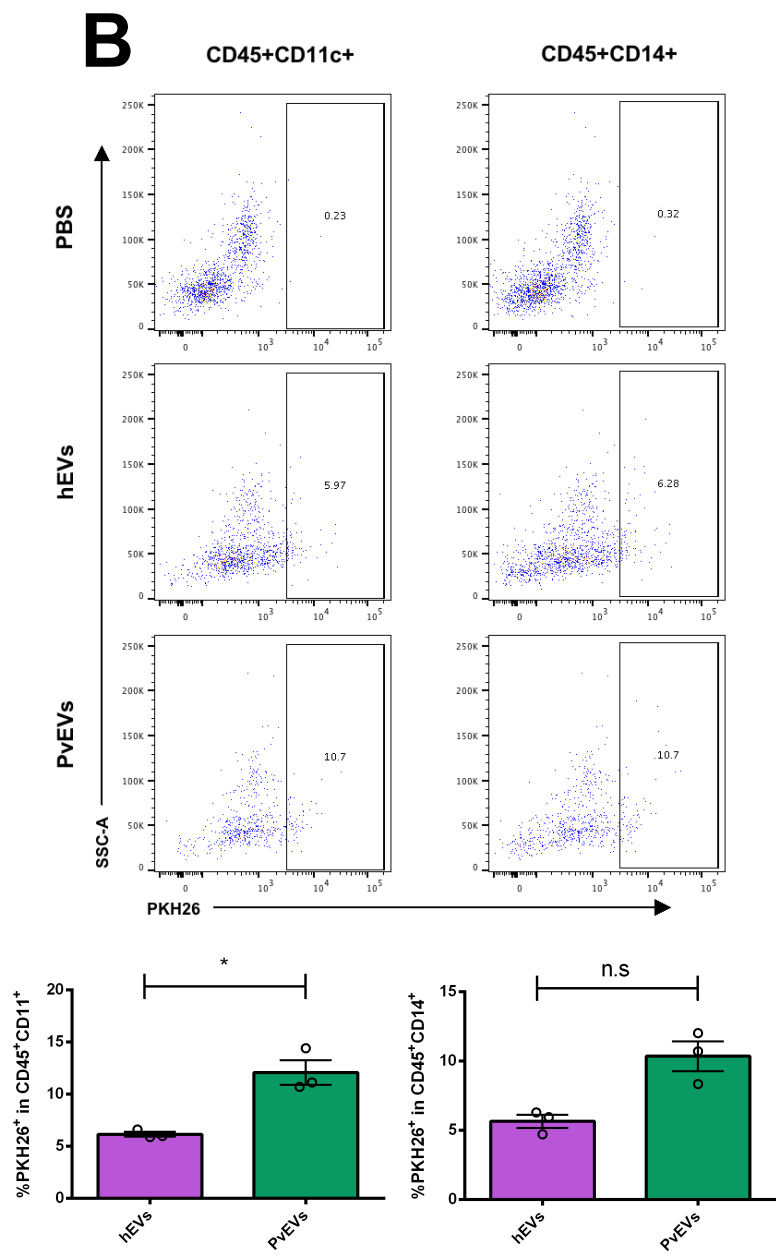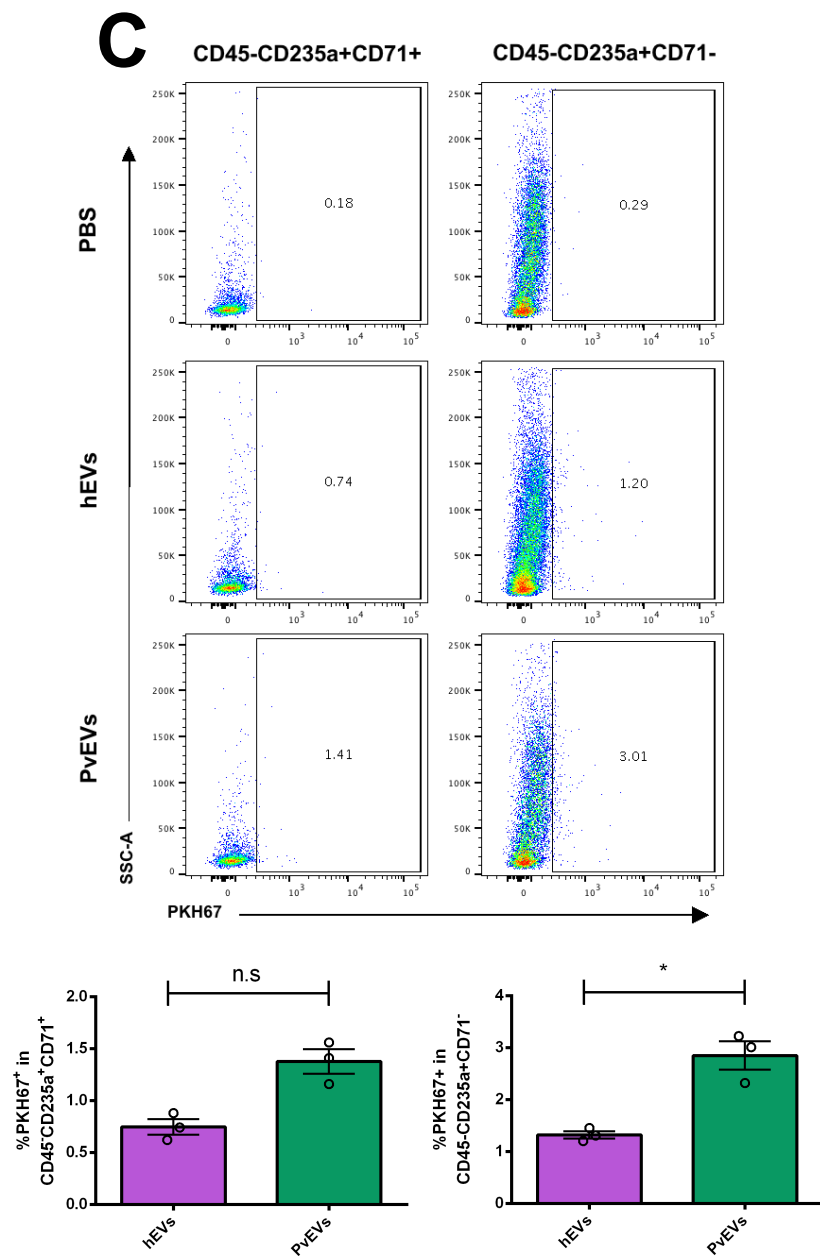

Supplement: Supplementary Data Sheet 5 — In vitro interaction of PvEVs and hEVs with total human spleen cells. Total spleen cells were obtained and frozen as described in the Methods section. Splenocytes were thawed and incubated with PKH26 labelled PvEVs, hEVs and PBS for 3h. Cells were washed and then stained with surface markers. Positive PKH26 staining was gated according to unlabeled cells. (A) Flow cytometry plots showing frequencies of NK, NKT, total T lymphocytes, CD4+ T cells and CD8+ T cells positive for PKH26. Frequencies quantification of three technical replicates is shown. (B) Flow cytometry plots of spleen monocytes and DCs showing frequencies of positive PKH26 cells. Frequencies quantification of three technical replicates is shown. (C) Flow cytometry plots of spleen reticulocytes and mature RBC showing frequencies of positive PKH26 cells. Frequencies quantification of three technical replicates is shown. Data shows a representative experiment of two different spleen donors analyzed independently. Statistical significance (P<0.05) was assessed using a Student’s t-test, *p<0.05. [file DataSheet_5.pdf]

## A T cell purification

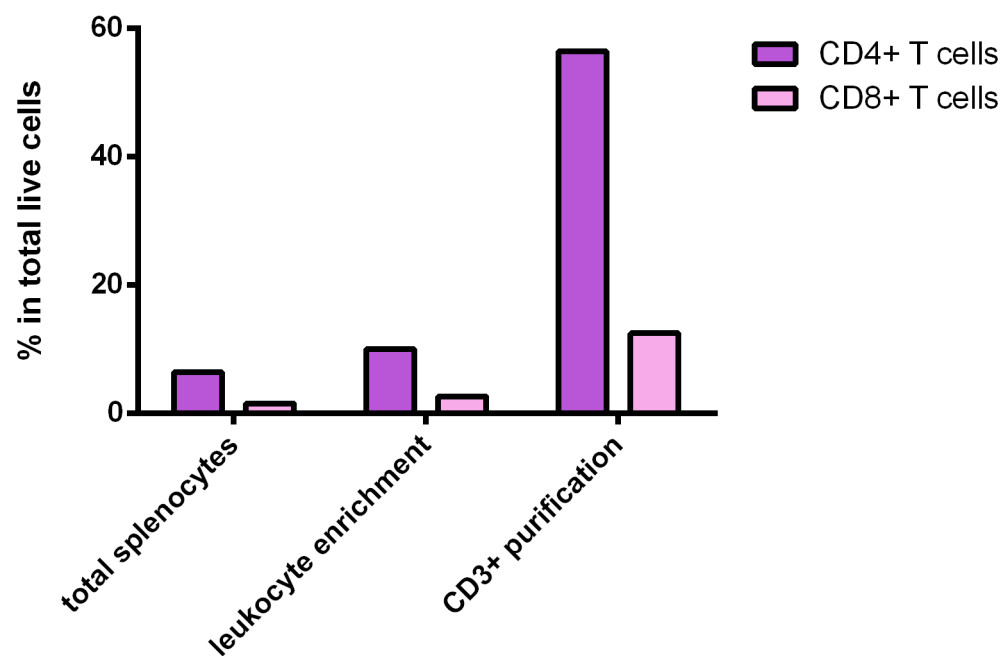

## B EVs interaction

### CD4+ T cells

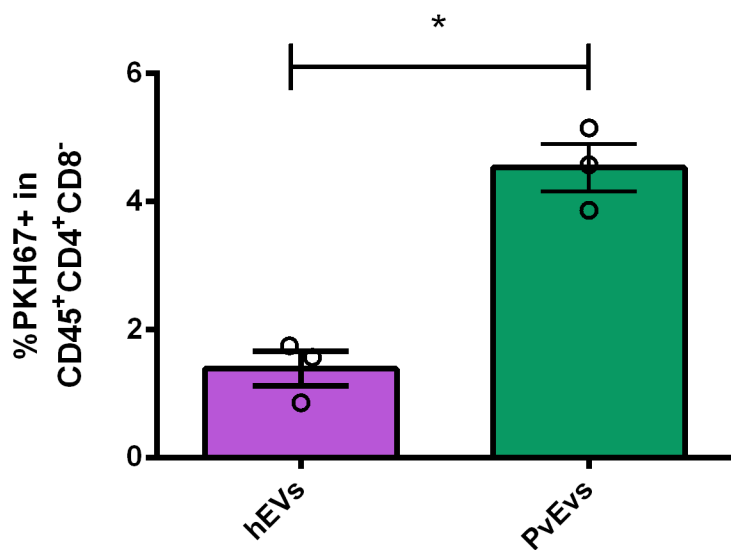

### CD8+ T cells

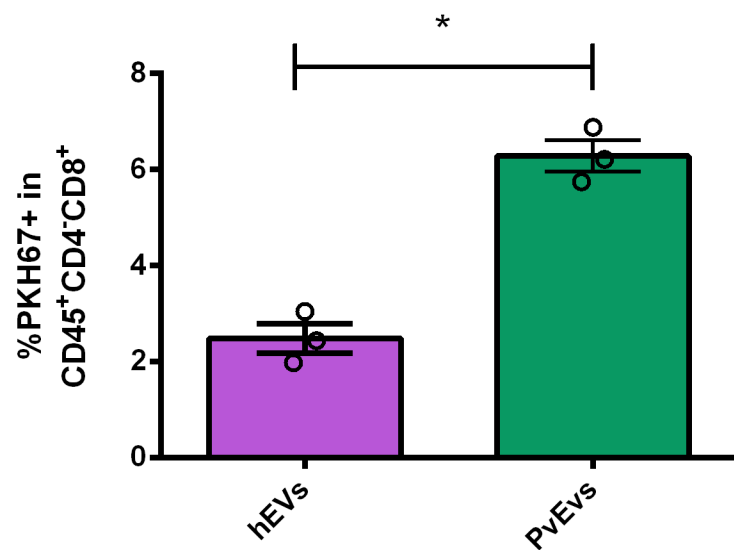

Supplement: Supplementary Data Sheet 6 — PvEVs and hEVs in vitro interaction with spleen T cells isolated by positive selection. (A) T cell purification. T cells were purified from spleen cell suspensions by a two-step procedure involving density gradient centrifugation and CD3+ immunomagnetic cell isolation. Cells from all purification steps were stained with fluorescent-conjugated antibodies against CD4 and CD8 surface markers and analyzed by flow cytometry. Plot corresponds to a representative image showing T cell enrichment quantification of two independent experiments. (B) PvEVs and hEVs in vitro interaction with spleen T cells. Frequencies of CD4+ and CD8+ T cells positively labeled with PKH26. PKH26 staining was gated according to cells incubated with PKH26-stained PBS. Frequencies quantification of three technical replicates is shown. Data is representative of two independent experiments performed with two spleen donors. Statistical significance (P<0.05) was assessed using a Student’s t-test, * p<0.05. [file DataSheet_6.pdf]

**A****DC enrichment**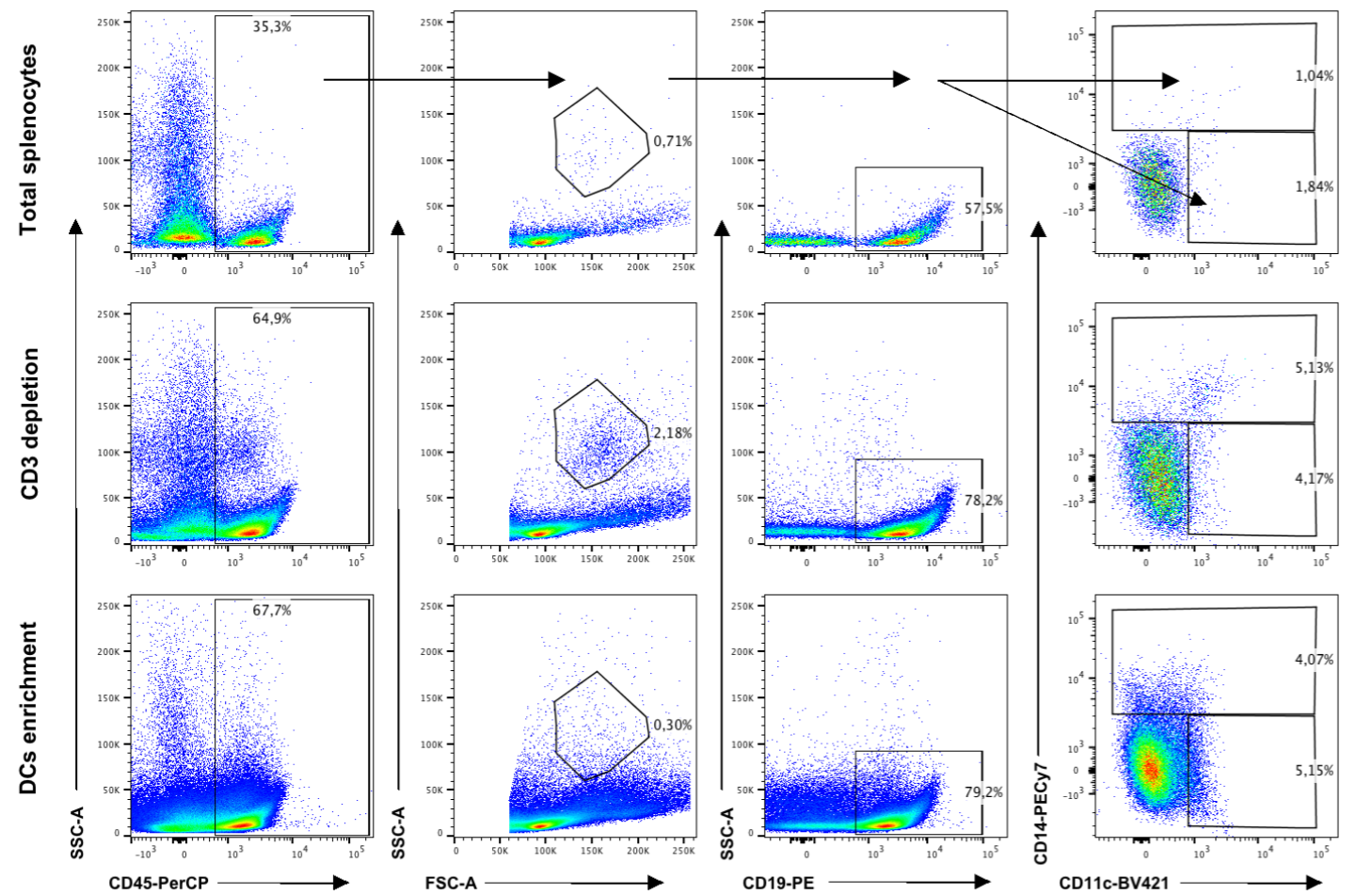**B****EVs interaction**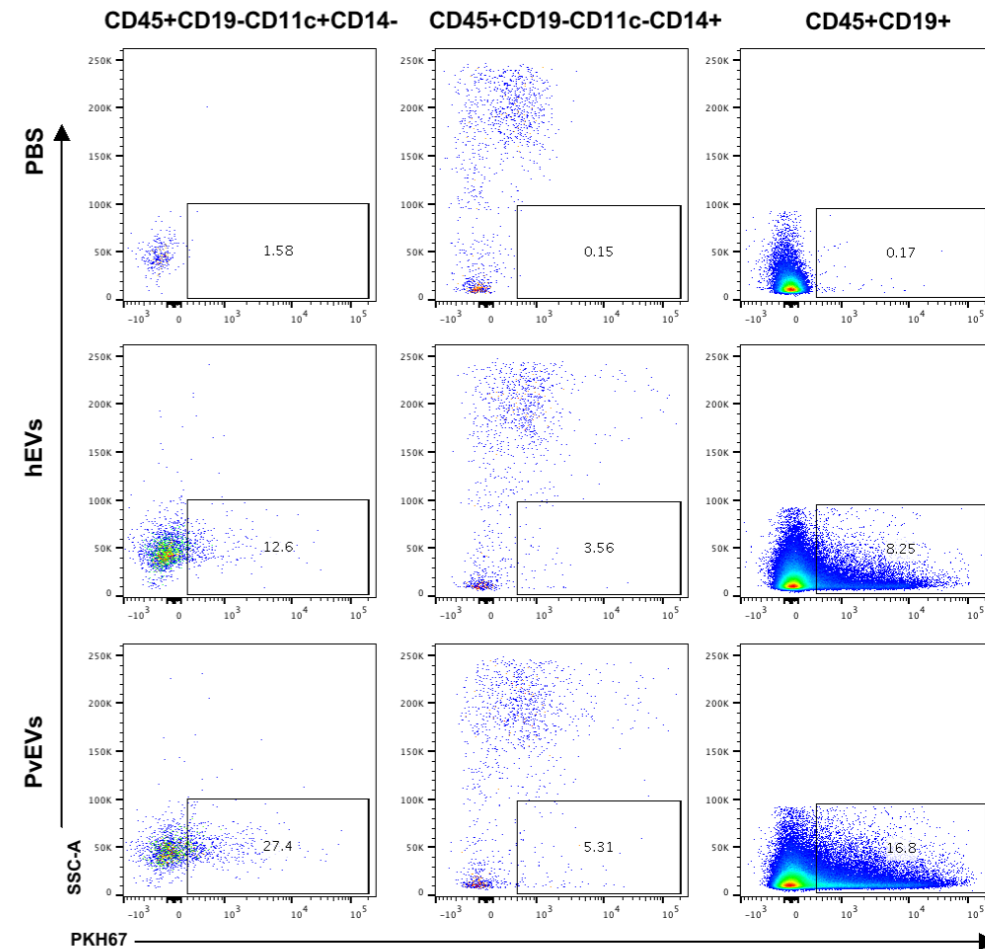**Total splenocytes**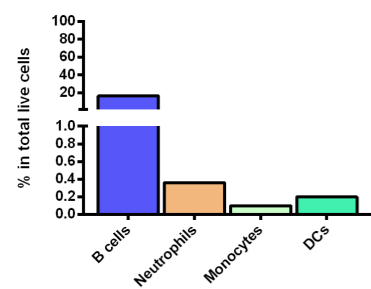**Ficoll CD3+ depletion**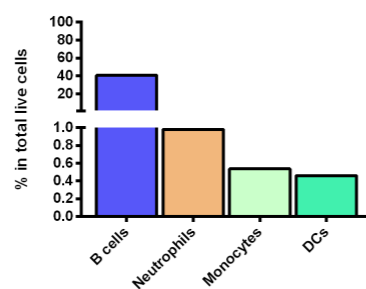**DCs enrichment**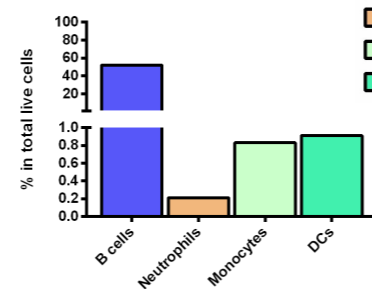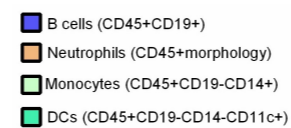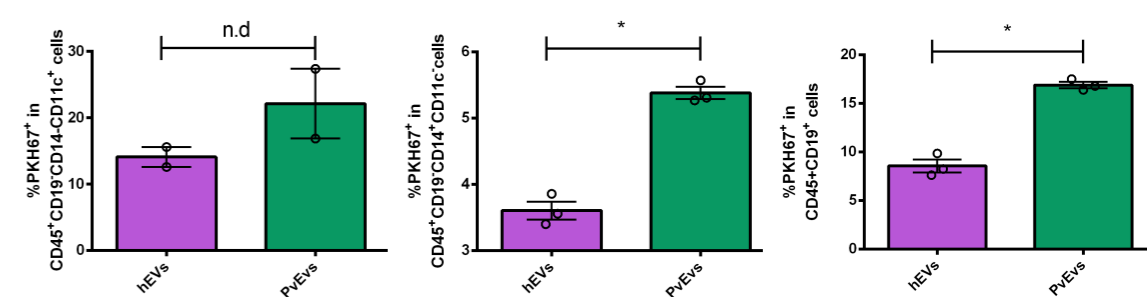

Supplement: Supplementary Data Sheet 7 — PvEVs and hEVs in vitro interaction with spleen phagocytic cells. (A) Dendritic cells enrichment. DCs were enriched from spleen cell suspensions by a two-step procedure as shown in Figure 2. Enriched cells were stained with fluorescent-conjugated antibodies against surface markers and analyzed by flow cytometry. Images show flow cytometry plots of the gating strategy to follow DC enrichment through the purification procedure. Enrichment quantification shown corresponds to one purification from one spleen donor. (B) PvEVs and hEVs in vitro interaction with spleen phagocytic cells. Flow cytometry plots showing frequencies of DCs, monocytes and B cells positively labeled with PKH67. PKH67 staining was gated according to cells incubated with PKH67-stained PBS. Frequencies quantification of three technical replicates is shown. Data represents mean and standard deviation of technical replicates of one experiment performed with one spleen donor. Statistical significance (P<0.05) was assessed using a Student’s t-test, *p<0,001. [file DataSheet_7.pdf]

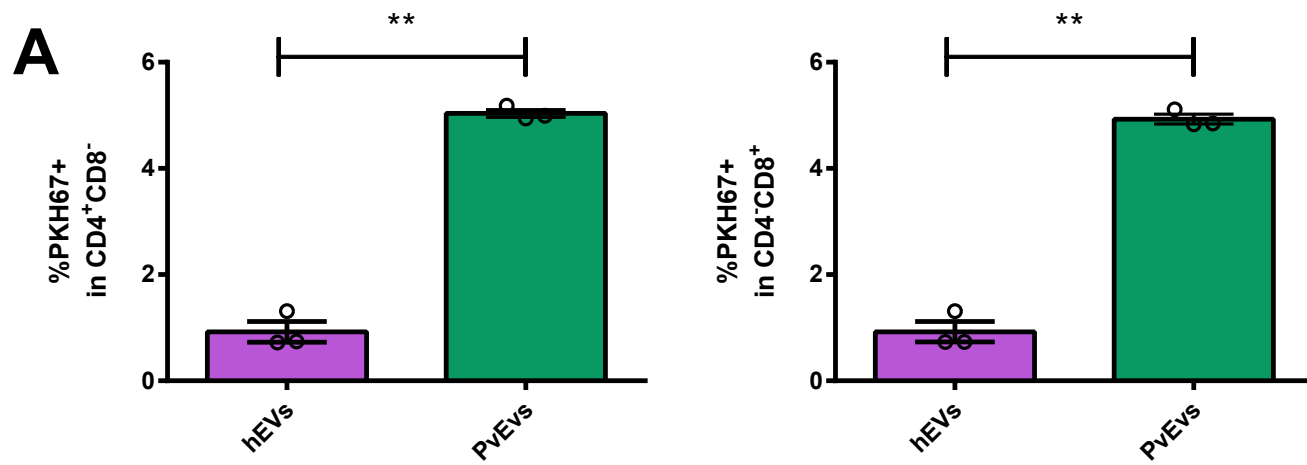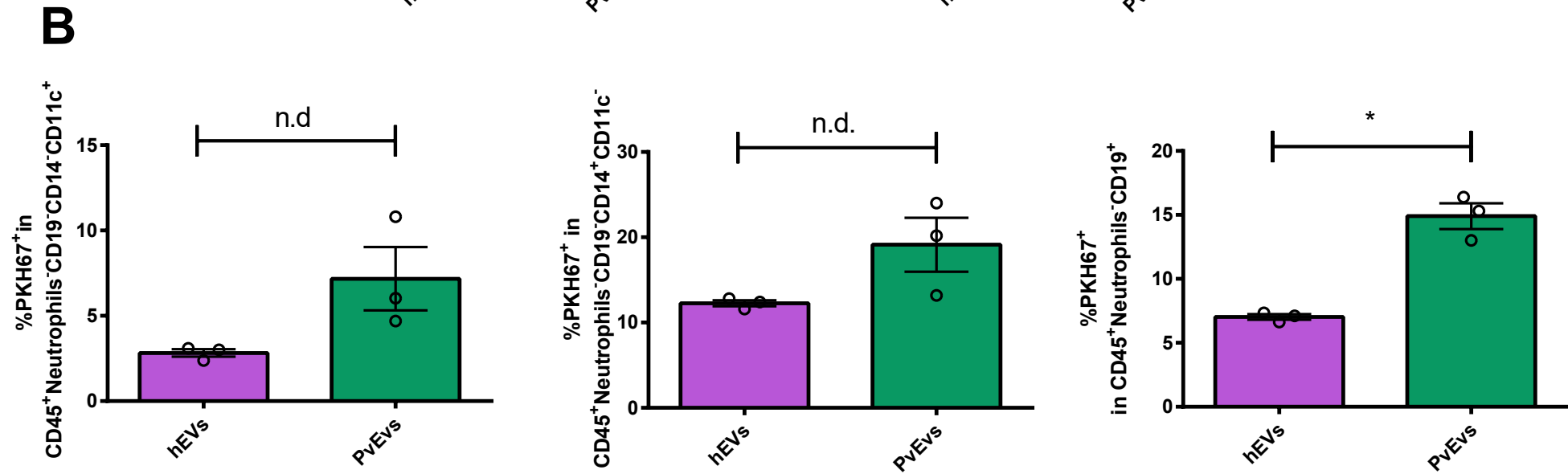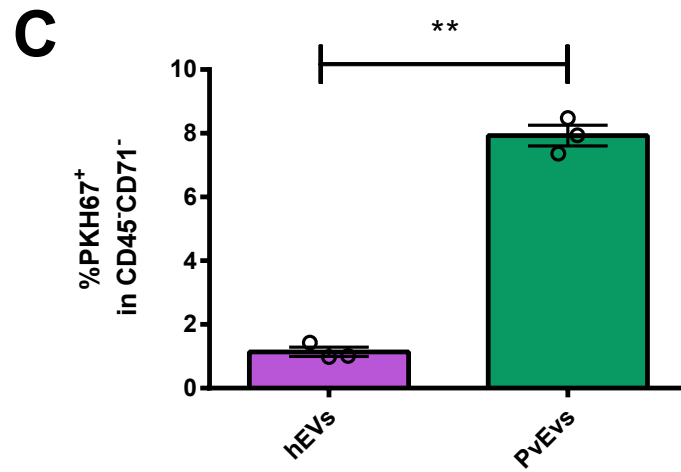

Supplement: Supplementary Data Sheet 8 — Summary of PvEVs and hEVs in vitro interaction with spleen cells from biological sample 2. (A) PvEVs and hEVs in vitro interaction with spleen T cells isolated by positive selection. T cells were purified from spleen cell suspensions by a two-step procedure involving density gradient centrifugation and CD3+ immunomagnetic cell isolation. Enriched cells were tested for its in vitro interaction with PKH67 labeled PvEVs and hEVs. Plots show frequencies of CD4+ and CD8+ T cells positively labeled with PKH26. (B) PvEVs and hEVs in vitro interaction with spleen phagocytic cells. Phagocytic cells were enriched by depletion of CD3+ cells. Cells were tested for its in vitro interaction with PKH67 labeled PvEVs and hEVs. Plots shows frequencies of DCs (CD45+Neutrophils-CD19-CD14-CD11c+), Monocytes (CD45+ Neutrophils-CD19-CD14+CD11c-) and B cells (CD45+ Neutrophils-CD19-) positively stained with PKH67. (C) PvEVs and hEVs in vitro interaction with spleen mature red blood cells (RBCs). RBCs were purified from spleen cell suspensions by a two-step procedure as shown in Figure 2. Enriched cells were tested for its in vitro interaction with PKH26 labeled PvEVs and hEVs. Plots shows frequencies of mature RBCs (CD45-CD71-) positively stained with PKH67. PKH67 was gated according to cells incubated with PKH67-stained PBS. All plots shows frequencies of three technical replicates. Statistical significance (P<0.05) was assessed using a Student’s t-test, *p<0,01, **p<0,001. [file DataSheet_8.pdf]
